# Phylogeography of the threatened tetraploid fish, *Schizothorax waltoni*, in the Yarlung Tsangpo River on the southern Qinghai-Tibet Plateau: implications for conservation

**DOI:** 10.1038/s41598-019-39128-y

**Published:** 2019-02-25

**Authors:** Xiang-Zhao Guo, Gui-Rong Zhang, Kai-Jian Wei, Wei Ji, Ruo-Jin Yan, Qi-Wei Wei, Jonathan P. A. Gardner

**Affiliations:** 1 Key Laboratory of Freshwater Animal Breeding, Ministry of Agriculture, College of Fisheries, Huazhong Agricultural University, Wuhan, 430070 P. R. China; 2 Freshwater Aquaculture Collaborative Innovation Center of Hubei Province, Wuhan, 430070 P. R. China; 3 School of Biological Sciences, Victoria University of Wellington, P O Box 600, Wellington, 6140 New Zealand; 4 Key Laboratory of Freshwater Biodiversity Conservation, Ministry of Agriculture, Yangtze River Fisheries Research Institute, Chinese Academy of Fishery Sciences, Wuhan, 430223 P. R. China; 5 Present Address: Guangdong Haid Group Co., Ltd., Guangzhou, 511400 P. R. China

## Abstract

The phylogeography of *Schizothorax waltoni*, an endemic and endangered tetraploid schizothoracine fish in the Yarlung Tsangpo River (YLTR) on southern margin of Qinghai-Tibet Plateau (QTP), was investigated using two mitochondrial DNA regions and eleven microsatellite loci. Analyses of concatenated sequences of cytochrome *b* (1141 bp) and the control region (712 bp) revealed high haplotype diversity and moderate nucleotide diversity. High genetic diversity was observed based on microsatellite variation. Both mtDNA and microsatellite analyses revealed significant genetic differentiation between the eastern population (Mainling) and the other four populations to the west, and non-significant genetic differentiation amongst the three central populations in the west. Significant genetic differentiation was observed between the western population (Shigatse) and the three central populations based on microsatellite analyses alone. Bayesian skyline plot analyses showed that *S. waltoni* experienced a pronounced population expansion 0.05 to 0.10 Ma. Hierarchical structure analyses of microsatellite data indicated that *S. waltoni* could be split into three groups (western, central and eastern YLTR). The results indicate that three management units should be considered for *S. waltoni*. Our findings highlight the need for the conservation and effective management of *S. waltoni*, which is a key member of the endemic and highly threatened fishes of the QTP.

## Introduction

Fishes are the most abundant group of vertebrates, and nearly half of their number are found in freshwater systems that constitute < 0.01% of the earth’s water^1^. Most freshwater systems now face multiple threats, all linked to anthropogenic activity, such that freshwater fish populations in many regions are endangered^2–4^. Indeed, anthropogenic effects are well documented in terms of localised and global extinctions of freshwater fishes on all human-inhabited continents^4–6^, leading to the suggestion that the world has already entered a sixth mass extinction event^7^.

Schizothoracine fishes (snow trout or snow minnows) consist of 15 genera and >100 species that are mainly distributed in Asia^8^. Of these, ~76 species and subspecies belonging to 11 genera occur on the Qinghai-Tibet Plateau (QTP) and its adjacent area^9^. Karyotype and DNA content analyses indicate that these fishes may be tetraploid, hexaploid or octoploid^10–12^. The genus *Schizothorax* includes 63 valid species^13^, of which ~40 species and subspecies are found in China^9,14^.

The Lhasa schizothoracin, *Schizothorax waltoni* Regan 1905 (Cyprinidae: Schizothoracinae), is an endemic tetraploid fish in the Yarlung Tsangpo River (YLTR) on the southern margin of the QTP^12,14,15^. Because limited information is available concerning population trends for this species, *S. waltoni* is assessed as being of Least Concern in the IUCN Red List of Threatened Species^16^. However, in the new Red List of China’s Vertebrates^17^, *S. waltoni* is assessed as Vulnerable. It is one of the most important commercial fishes of this region and is fished mainly by set gill nets and electrofishing. It is long-lived, cold-adapted, has a slow growth rate, late sexual maturity and low fecundity^9,18^, and is therefore especially vulnerable to human activities such as overfishing, biological invasion by exotic fishes and construction of hydroelectric dams^18–20^.

Before the 1950s, schizothoracine fishes in Tibet were indirectly protected due to Tibetan religious belief, with restrictions on fishing and fish eating being imposed by the supreme Tibetan religious authority^21^. However, with the increase of immigrants and tourists from inland areas and the rising demand for fish and fisheries products, overexploitation and illegal fishing are now serious threats to the long-term survival of schizothoracine fishes in Tibet^18,19^. Concurrently, many non-native species (e.g. goldfish (*Carassius auratus*) and topmouth gudgeon (*Pseudorasbora parva*)) have been introduced into the YLTR and are now out-competing and displacing the endemic schizothoracine fishes^19,20^. In addition, ongoing construction of hydroelectric dams has contributed substantially to habitat degradation for endemic fishes. As a consequence, *S. waltoni* is now threatened by sharp declines in population sizes^19^. Conservation of the natural populations of *S. waltoni* is now of primary concern, in particular given the limited geographic distribution of this species.

Despite the ecological and fisheries importance of *S. waltoni*, little is known about the genetic diversity and genetic structure of its populations, and the information that does exist is derived indirectly from phylogenetic analyses rather than from population genetic analyses. Understanding the level of genetic diversity and population genetic structure of *S. waltoni* is important for the effective conservation (e.g. hatchery production of juveniles from appropriate stocks) and management (e.g. restrictions on fishing activities) of the species.

In the present study, we used mitochondrial DNA (mtDNA) cytochrome *b* gene (Cyt *b*) and control region (CR) sequencing and an assessment of eleven polymorphic microsatellite loci to evaluate genetic diversity and genetic differentiation within and amongst populations, and to explore conservation management strategies for this species. The challenge faced by a study such as this when working on a threatened species with limited geographic distribution is to sample individuals from sufficient populations and across as broad a geographic range as possible. The results of this study provide new insights into the population genetic structure of this threatened fish that may contribute to conservation, management and sustainable utilisation of this important species, and which may also help to provide context for other threatened endemic fish species in the same or similar river systems.

## Results

### Genetic diversity and genetic structure of five populations - mtDNA sequence data

**Figure 1:**
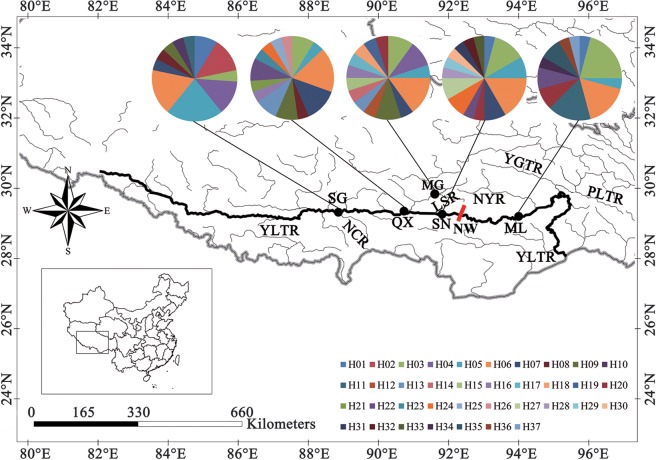
Sampling locations and geographical distribution of 37 Cyt *b* + CR haplotypes identified in *Schizothorax waltoni* on the Qinghai-Tibet Plateau. YLTR, Yarlung Tsangpo River; PLTR, Parlung Tsangpo River; YGTR, Yigong Tsangpo River; NCR, Nianchu River; LSR, Lhasa River; NYR, Nyang River; NW, Nierikar Waterfall. SG – Shigatse; MG – Maldrogongkar; QX – Quxu; SN – Shannan; ML – Mainling. The map was drawn using ArcGIS 10.2 (ESRI, CA, USA) and Adobe Photoshop CS5.1 (Adobe Systems Inc., CA, USA).

Both mtDNA Cyt *b* and CR were amplified and sequenced from 114 samples of *S. waltoni*. Full Cyt *b* sequences 1141 bp exhibited 21 variable sites, 17 of which were parsimony informative. Partial CR sequences (712 bp) had 28 variable sites, 22 of which were parsimony informative, with no indels. There were 17 Cyt *b* haplotypes and 28 CR haplotypes defined, and their sequences were submitted to GenBank (Accession numbers MK243405–MK243421 for Cyt *b* and MK243422–MK243449 for CR). Cyt *b* and CR sequences from each sample were concatenated into one sequence of 1853 bp (5′-Cyt *b* + CR-3′) for phylogenetic analysis, to give a total of 37 mtDNA haplotypes in the concatenated data set. Three haplotypes (H03, H05 and H06) were shared amongst the five populations (Fig. 1; Table S1); each population was characterised by one or more private haplotypes. The five populations were characterised by high haplotype diversity (*Hd* = 0.941) and moderate nucleotide diversity (*π* = 0.0052) values, respectively (Table 1).

**Table 1:** Genetic diversity indices for five populations of *Schizothorax waltoni* based on the Cyt *b*, CR and concatenated Cyt *b* + CR sequence data set.

Sequence	Population	*n*	*S*	*h*	*Hd*	*K*	*π*
Cyt *b*	SG	23	12	7	0.877	3.478	0.0031
	MG	20	15	10	0.900	3.579	0.0031
	QX	23	14	8	0.818	3.360	0.0029
	SN	24	14	10	0.891	4.072	0.0036
	ML	24	13	7	0.819	4.058	0.0036
	Total	114	21	17	0.877	3.937	0.0035
CR	SG	23	19	10	0.897	5.190	0.0073
	MG	20	22	14	0.947	5.284	0.0074
	QX	23	17	12	0.937	5.289	0.0074
	SN	24	21	10	0.880	5.493	0.0077
	ML	24	20	10	0.895	5.594	0.0079
	Total	114	28	28	0.918	5.597	0.0079
Cyt *b* + CR	SG	23	31	11	0.910	8.668	0.0047
	MG	20	37	15	0.968	8.863	0.0048
	QX	23	31	14	0.949	8.648	0.0047
	SN	24	35	15	0.953	9.565	0.0052
	ML	24	33	11	0.909	9.652	0.0052
	Total	114	49	37	0.941	9.534	0.0052

*n*, number of samples, *h*, number of haplotypes; *S*, number of segregating sites; *Hd*, haplotype diversity; *π*, nucleotide diversity; *K*, number of nucleotide differences.

All pairwise *Φ*_ST_ values between ML (eastern region) and the other populations (SG, MG, QX, SN, western region) were significantly different from zero after FDR correction (Table 2).

**Table 2:** Pairwise *Φ*_ST_ (below diagonal), average Kimura 2-parameter (K2P) genetic distance values between pairs of populations (above diagonal) based on the Cyt *b*, CR and concatenated Cyt *b* + CR sequence data set.

Sequence	Population	SG	MG	QX	SN	ML
Cyt *b*	SG	—	0.003	0.003	0.003	0.004
	MG	−0.038	—	0.003	0.003	0.004
	QX	−0.021	−0.033	—	0.003	0.004
	SN	−0.017	−0.029	−0.023	—	0.004
	ML	**0.179**	**0.160**	**0.195**	**0.139**	—
CR	SG	—	0.007	0.007	0.007	0.009
	MG	−0.035	—	0.007	0.007	0.009
	QX	−0.026	−0.031	—	0.007	0.009
	SN	−0.026	−0.032	−0.023	—	0.009
	ML	**0.154**	**0.134**	**0.156**	**0.104**	—
Cyt *b* + CR	SG	—	0.005	0.005	0.005	0.006
	MG	−0.036	—	0.005	0.005	0.006
	QX	−0.024	−0.032	—	0.005	0.006
	SN	−0.022	−0.030	−0.023	—	0.006
	ML	**0.164**	**0.145**	**0.172**	0.119	—

Significant *Φ*_ST_ values are in bold after FDR testing (*P* < 0.05).

For the concatenated mtDNA Cyt *b* + CR sequences, AMOVA indicated that significant difference existed amongst populations (*P* < 0.01), but not between regions (Table 3). The K2P genetic distances and pairwise *Φ*_ST_ values between populations ranged from 0.005 to 0.006 and from −0.036 to 0.172, respectively (Table 2). The K2P genetic distances between the groups (western (SG) vs central (QX, MG, SN); western versus eastern (ML); central versus eastern) were 0.005, 0.006, and 0.006, respectively. Based on the Cyt *b* sequences or the CR sequences alone, similar results were observed for the AMOVA analysis, K2P distances and pairwise *Φ*_ST_ values (Tables 2 and 3).

**Table 3:** Analysis of molecular variance (AMOVA) of *Schizothorax waltoni* population genetic variation based on Cyt *b*, CR and concatenated Cyt *b* + CR sequence data set, as well as on nuclear DNA microsatellite variation (based on 266 microsatellite bands).

Source of variation	df	Variance component	Percentage of variation	Fixation index
**Cyt** ***b***				
Among populations	4	0.135	6.76	*Φ*_ST_ = 0.676***
Within populations	109	1.860	93.24	—
Between regions (west/east)	1	0.439	19.52	*Φ*_CT_ = 0.195
Among populations within regions	3	−0.048	−2.13	*Φ*_SC_ = −0.026
Within populations	109	1.860	82.61	*Φ*_ST_ = 0.174***
**CR**				
Among populations	4	0.138	4.87	*Φ*_ST_ = 0.049**
Within populations	109	2.688	95.13	—
Between regions (west/east)	1	0.511	16.35	*Φ*_CT_ = 0.164
Among populations within regions	3	−0.075	−2.40	*Φ*_SC_ = 0.029
Within populations	109	2.688	86.05	*Φ*_ST_ = 0.140**
**Cyt** ***b*** ** + CR**				
Among populations	4	0.272	5.65	*Φ*_ST_ = 0.057**
Within populations	109	4.548	94.35	—
Between regions (west/east)	1	0.950	17.68	*Φ*_CT_ = 0.177
Among populations within regions	3	−0.123	−2.28	*Φ*_SC_ = −0.028
Within populations	109	4.548	84.61	*Φ*_ST_ = 0.154**
**Microsatellites**				
Among populations	4	0.989	5.86	*F*_ST_ = 0.059***
Within populations	216	15.870	94.14	—
Between regions (west/east)	1	1.495	8.37	*F*_CT_ = 0.084
Among populations within regions	3	0.489	2.74	*F*_SC_ = 0.030***
Within populations	216	15.870	88.89	*F*_ST_ = 0.111***

**P < 0.01, ***P < 0.001.

**Figure 2:**
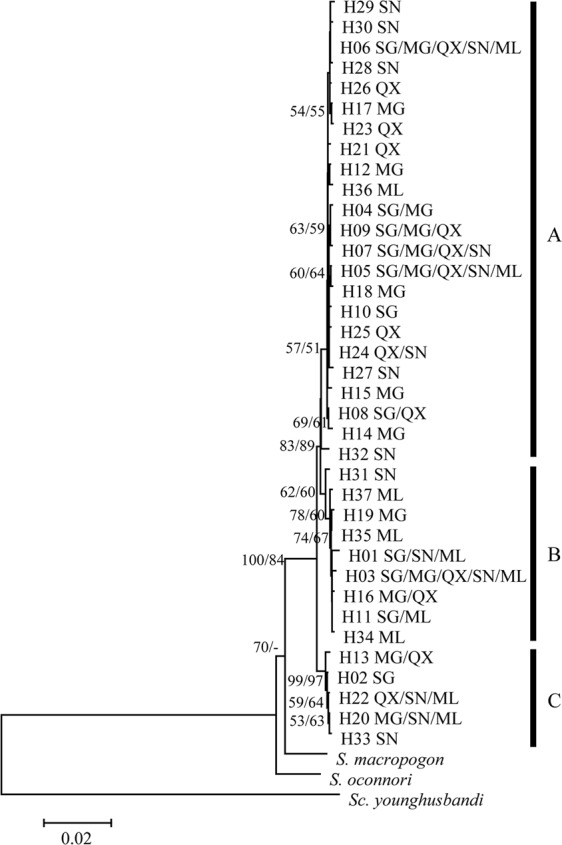
The neighbour-joining phylogenetic tree of *Schizothorax waltoni*, based on concatenated Cyt *b* + CR sequence haplotypes (1853 bp). The haplotypes provided at each twig and their geographical locations are shown in Table S3 and Fig. 1. The numbers above the branches correspond to bootstrap support values > 50% obtained in the NJ/ML analyses, respectively.

**Figure 3:**
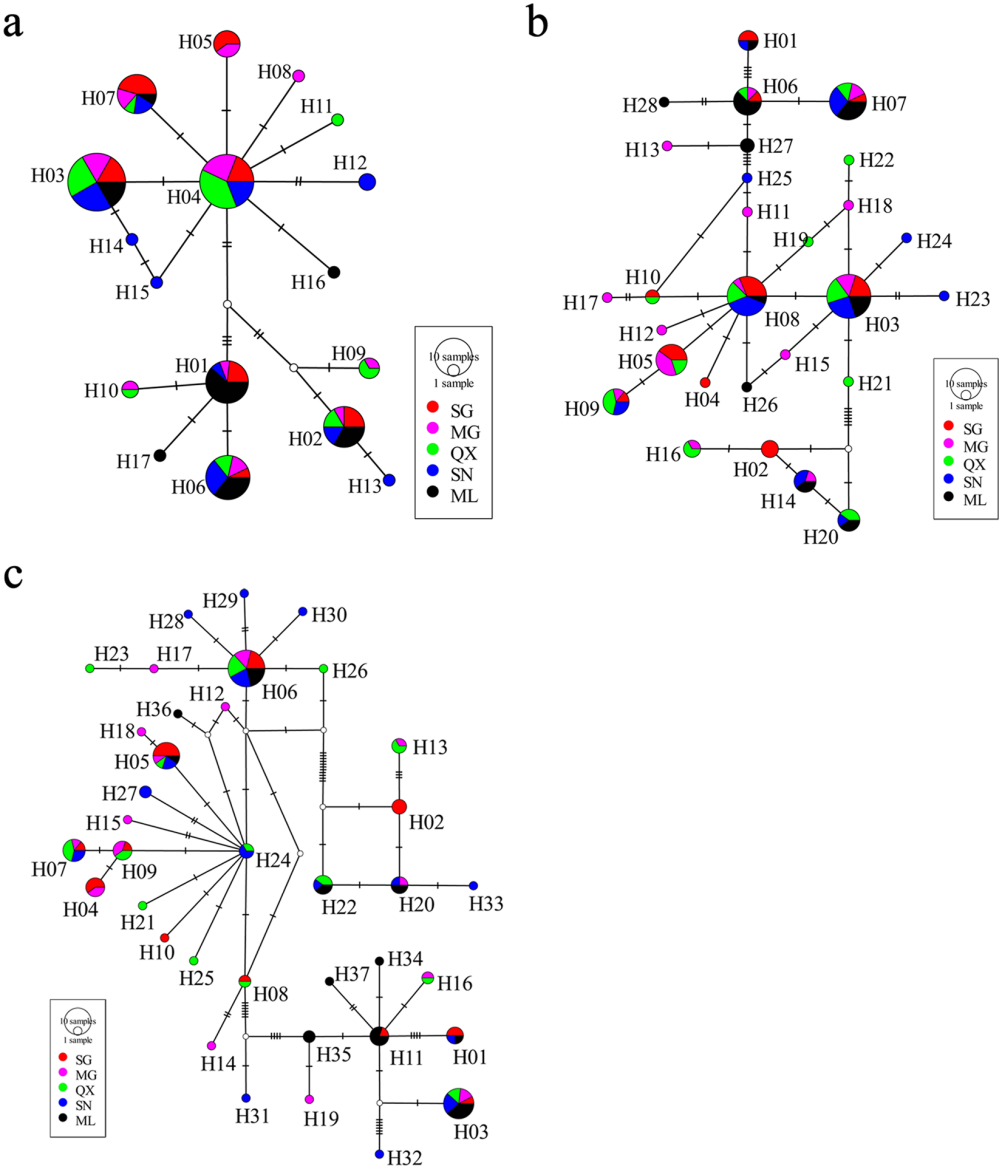
Median-joining network of mtDNA (**a**) Cyt *b* haplotypes, (**b**) CR haplotypes and concatenated (**c**) Cyt *b* + CR sequence haplotypes from five populations of *Schizothorax waltoni*. The size of each haplotype circle denotes the number of observed individuals. Colours correspond to different regions. White circles represent intermediate haplotypes not observed. Small bars across lines represent base pair steps between adjacent haplotypes.

**Figure 4:**
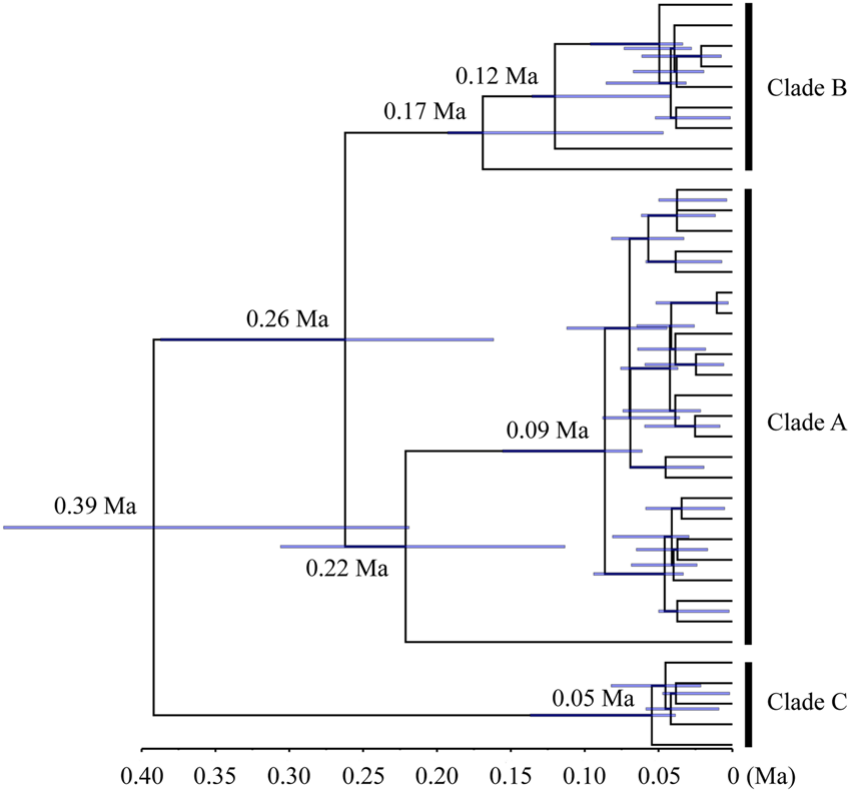
Bayesian phylogenetic tree for *Schizothorax waltoni*, based on concatenated mtDNA sequences (Cyt *b* + CR) haplotypes. The numbers above the branches are the estimates of divergence times (million years, Ma) within *Schizothorax waltoni* for the major nodes by BEAST analysis. Blue shaded bars indicate the 95% highest posterior density (HPD) for node ages and scale bar represents time in millions of years from the present day.

The two phylogenetic methods (NJ and ML) yielded trees with very similar topologies based on concatenated mtDNA (only the NJ tree with bootstrap values from the NJ and ML trees is shown – Fig. 2). The 37 haplotypes formed two distinct clades that were well supported by bootstrap values. However, no clear pattern of geographic structure was identified, consistent with the AMOVA results (Table 3). The NJ and ML phylogenetic analyses exhibited similar results based on Cyt *b* or CR alone (Figs S1 and S2). Similar to the NJ and ML results, median-joining networks showed that all haplotypes from the five populations did not reveal a distinct geographical structure. No obvious star burst-like topological structure or central haplotypic distribution was observed in the network maps (Fig. 3). The divergence time amongst the main clades estimated based on the combined mtDNA Cyt *b* + CR and on the Cyt *b* data sets were 0.26 Ma–0.39 Ma and 0.33 Ma–0.44 Ma, respectively (Figs 4, S3).

**Figure 5:**
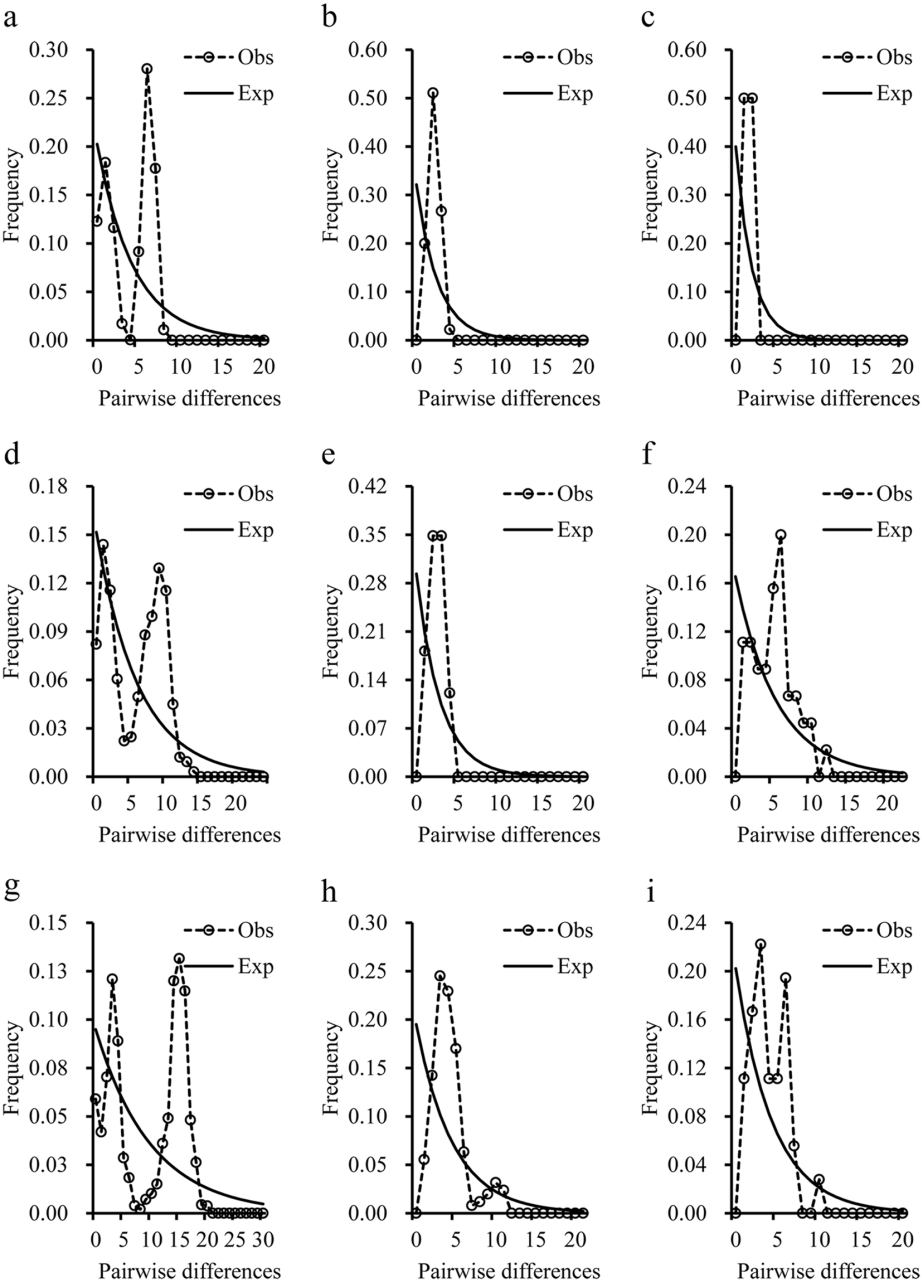
Observed and expected mismatch distributions for *Schizothorax waltoni* in the five populations. Cyt *b* sequences: (**a**) total haplotypes, (**b**) clade A, (**c**) clade C; CR sequences: (**d**) total haplotypes, (**e**) clade A, (**f**) clade B; Cyt *b* + CR sequences: (**g**) total haplotypes, (**h**) clade A, (**i**) clade B; Dashed line, observed distribution; Solid line, theoretical expected distribution under a constant population size model.

Multimodal mismatch distributions and non-significant Tajima’s *D* and Fu’s *F*s values indicated that *S. waltoni* has remained relatively stable over time in all five populations (Fig. 5a,d,g; Table S2). However, unimodal mismatch distributions, significant negative Tajima’s *D*, Fu’s *F*s, non-significant *SSD* and *r* values were found in the haplotype clade A of three NJ trees (Fig. 5b,e,h; Table S2). All these results supported a sudden population expansion model for clade A in each NJ tree.

**Figure 6:**
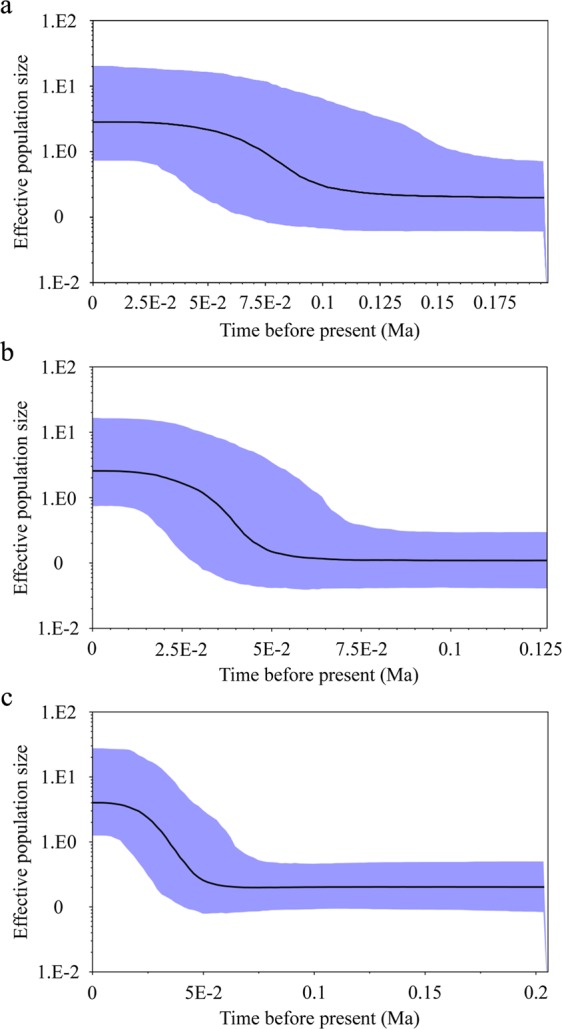
Bayesian skyline plots (BSPs) of *Schizothorax waltoni*. (**a**) Cyt *b*; (**b**) CR; (**c**) Cyt *b* + CR; The *X*-axis shows time in millions of years before present. The *Y*-axis (logarithmic scale) indicates effective population size of females (*N*_e_) estimates multiplied by generation time (T_gen_). The solid line indicates the median of population size, and the 95% highest posterior density (HPD) interval is depicted in blue.

Bayesian skyline plot (BSP) analyses supported the hypothesis of relatively recent population expansion of clade A (Figs 6a–c and S4a–d). BSP revealed that the population size had no pronounced demographic changes for a long time, before it experienced a pronounced population expansion at ~0.05 Ma–0.10 Ma (Figs 6a–c, S4a–d).

### Genetic diversity and genetic structure of five populations - microsatellite data

As expected for a tetraploid, each microsatellite locus had up to 4 amplified bands per individual (Table S3), and all 11 microsatellite loci were highly polymorphic. In total, 266 bands from 11 microsatellite loci were amplified in 221 fish (Table 4). Number of bands per locus ranged from 5 (loci LLK28 and Scho23) to 62 (locus Scho24) (Table S3). Genetic diversity index values were consistent in pattern across all five populations (Table 4). Total number of bands for each population ranged from 135 (ML) to 180 (MG). Analysis of putative outlier-*F*_ST_ loci showed that 11 bands (BayesScan), 11 bands (Arlequin) and 15 bands (Mcheza) were detected as outlier candidates to be under selection (Fig. S5; Table S4). However, only nine common bands were identified as outlier loci by any two of the three methods (Table S4). The nine outlier bands were removed and further analyses were based on the 257 putatively neutral loci. All results described below are from analyses of both the full data set (all 266 bands) and the reduced data set (257 putatively neutral bands). Of the 266 bands, 199 bands (74%) were shared by the five populations, and the other 67 bands were population-specific i.e., private alleles (Table 4). Population ML had lower genetic diversity as revealed by percentage of polymorphic loci (*PPL*), Nei’s gene diversity (*H*) and Shannon’s information index (*I*) compared to the other four populations (Table 4). Similar results were observed for the 257 band data set (Table S5).

**Table 4:** Nuclear DNA diversity indices for five populations of *Schizothorax waltoni* based on analysis of 266 microsatellite bands.

Population	Total bands	Private bands	*PPL* (%)	*H*	*I*
SG	173	24	64.29	0.080	0.143
MG	180	15	66.54	0.082	0.147
QX	163	5	60.53	0.080	0.142
SN	167	10	62.03	0.079	0.141
ML	135	13	49.62	0.073	0.128
Mean	163.6	13.4	60.60	0.079	0.140
Total	266	67	—	—	—

*PPL*, percentage of polymorphic loci; *H*, Nei’s gene diversity index; *I*, Shannon’s information index.

**Figure 7:**
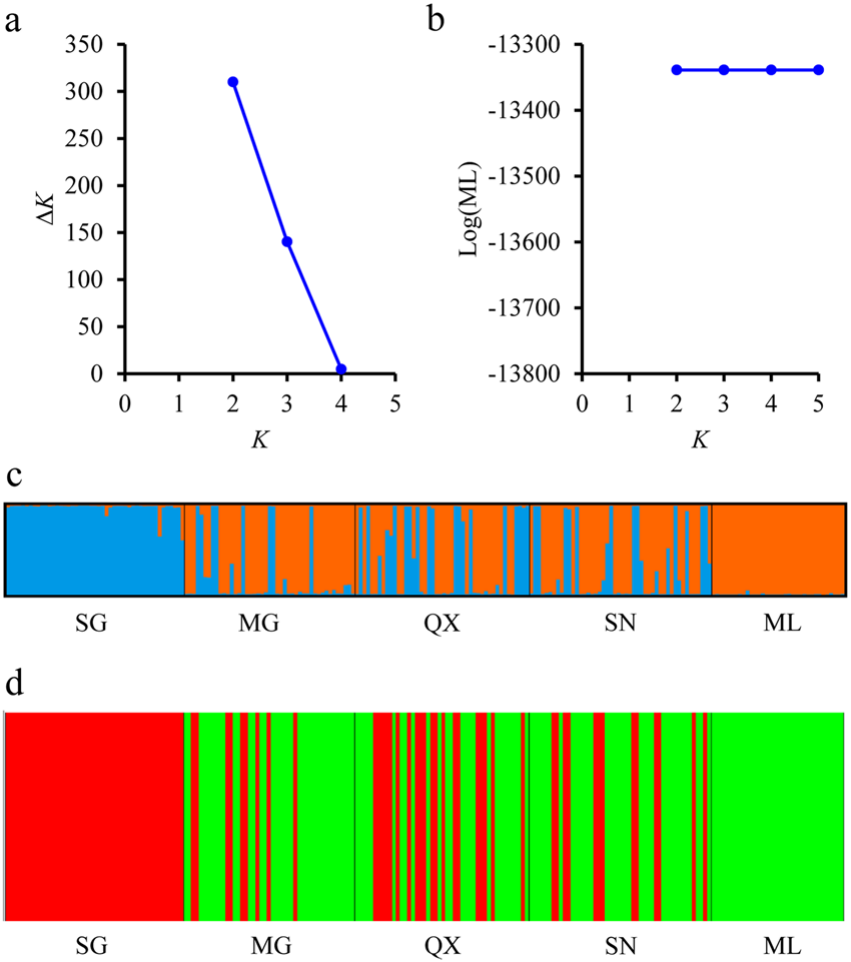
Cluster analyses of *Schizothirax waltoni* populations based on all 266 amplified bands at 11 microsatellite loci. (**a**) Inference of best *K* in STRUCTURE 2.3; (**b**) Inference of best *K* in BAPS 6.0; (**c**) Histogram of the assignment test using STRUCTURE 2.3 (*K* = 2); (**d**) Histogram of the assignment test using BAPS 6.0 (*K* = 2); Each individual is represented by a vertical coloured line, and each color corresponds to a genetic cluster.

**Figure 8:**
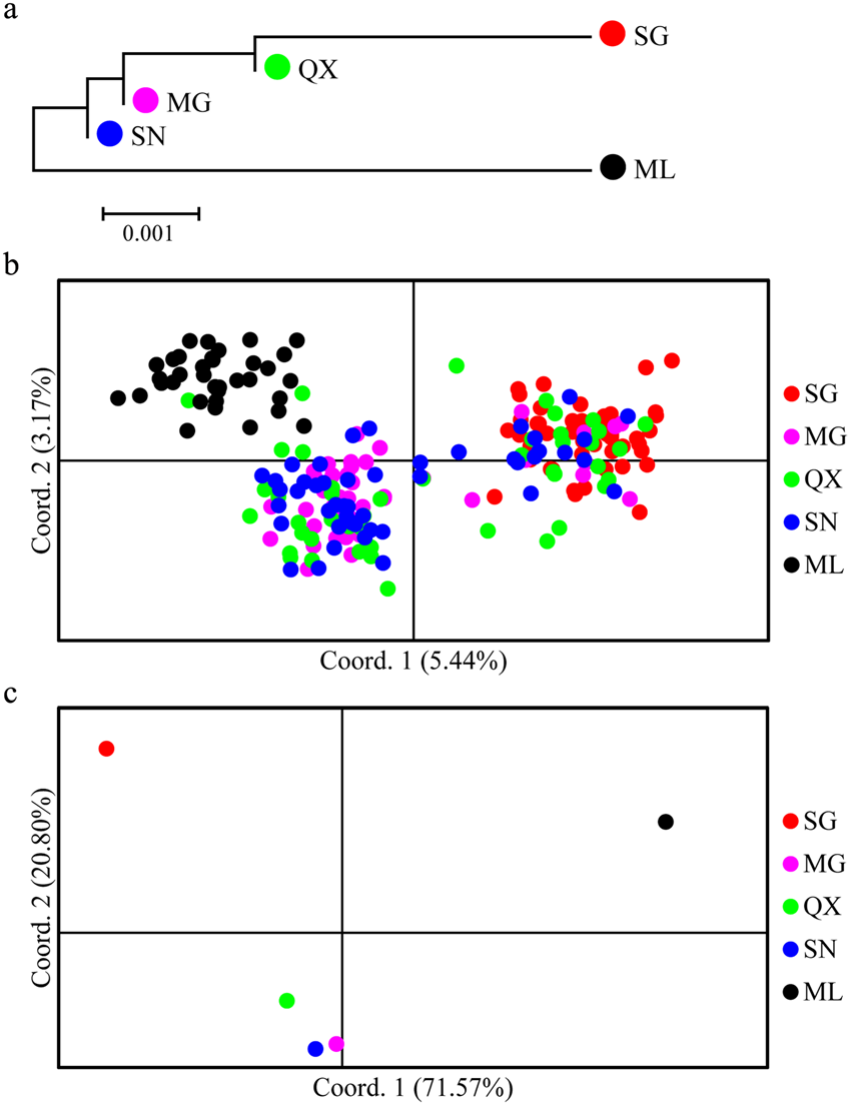
Genetic relationship amongst five populations of *Schizothorax waltoni* based on all 266 amplified bands at 11 microsatellite loci. (**a**) NJ clustering tree based on the Nei’s unbiased genetic distances amongst populations; (**b**) Principal coordinate analysis (PCoA) based on the pairwise genetic distances between individuals; (**c**) PCoA based on the pairwise genetic distances between populations.

Based on the full 266 band data set, all pairwise *F*_ST_ values were significantly different from zero after FDR correction, except the comparisons amongst the three geographically central populations (MG, QX and SN) (Table 5). AMOVA results indicated that significant differences existed amongst populations and amongst populations within regions (*P* < 0.01), but not between regions (Table 3). Nei’s unbiased pairwise genetic distance values and pairwise *F*_ST_ values ranged from 0 to 0.013 and from 0.001 to 0.163, respectively (Table 5). STRUCTURE and BAPS analyses indicated that the Δ*K* and Log(ML) statistics reached their largest value at *K* = 2, respectively, suggesting that there were two subgroups in the five *S. waltoni* populations (Fig. 7a–d). These were composed of fishes from SG at the upstream (western) end of the YLTR and the fishes from ML at the downstream (eastern) end of the river. The fishes from the three geographically central populations fell into an intermediate position between these two groups. The NJ clustering tree based on the Nei’s unbiased genetic distances amongst populations (Fig. 8a) and the PCoA plots of the relationships amongst individuals (Fig. 8b) and amongst populations (Fig. 8c) were consistent with the pattern of the results of STRUCTURE and BAPS analyses, and suggested that the ML population was the most genetically differentiated and therefore most distinct. Results based on the reduced data set were all similar to those described above (Tables S6 and S7; Figs S6a,c; S7a,c), except that no clear population genetic structure was found based on BAPS (Fig. S6b,d) and PCoA analysis amongst individuals (Fig. S7b).

**Table 5:** Matrix of pairwise *F*_ST_ values (below diagonal) and Nei’s unbiased genetic distances (above diagonal) amongst five *Schizothorax waltoni* populations based on variation at 266 microsatellite bands.

Population	SG	MG	QX	SN	ML
SG	—	0.004	0.003	0.004	0.013
MG	**0.055**	—	0.001	0.000	0.006
QX	**0.042**	0.004	—	0.001	0.007
SN	**0.060**	0.001	0.006	—	0.006
ML	**0.163**	**0.092**	**0.102**	**0.097**	—

Significant *F*_ST_ values are in bold after FDR testing (*P* < 0.05).

### Isolation by distance (IBD)

Mantel tests revealed no significant correlation between *Φ*_ST_/(1 − *Φ*_ST_) and Ln(*d*) for mtDNA sequence variation (*R*^2^ = 0.59, *P* = 0.097, Fig. S8a). However, significant correlation was observed between *F*_ST_/(1 − *F*_ST_) and Ln(*d*) based on microsatellite variation (*R*^2^ = 0.74, *P* = 0.043, Fig. S8b). The results confirmed the occurrence of an IBD model of gene flow, consistent with the results of the STRUCTURE analysis. When IBD analysis was performed on the reduced data set no significant correlation was observed (*R*^2^ = 0.69, *P* = 0.078, Fig. S9).

## Discussion

As the largest high-elevation ecosystem in the world, the Qinghai-Tibet Plateau (QTP) is characterised by high species richness and abundant endemic species, and has been identified as one of the most important centres of biodiversity in the world^22^. The unique status of the QTP arises because of the influence of its geological history on its biodiversity^23,24^. The QTP has, however, been identified as a region in need of urgent protection to conserve its biodiversity because of the many anthropogenic threats that it now faces^25^. Whilst conservation measures have been put in place to protect some QTP freshwater fishes (the Basum-Tso National Aquatic Germplasm Resources Conservation Area in Tibet was established in 2011 by the Ministry of Agriculture of China) and *S. waltoni* in particular (e.g., a breeding and restocking programme), such actions are unlikely to safeguard the long-term future of this species. Whilst identifying threats to freshwater fish biodiversity may be relatively straight forward and have been well documented^2^, the establishment of strategies to mitigate threats that occur at global scales (e.g., climate change) is a much harder prospect, and is the real challenge now facing conservation biology.

Like other polyploid schizothoracine fishes in Tibet^26^, *S. waltoni* exhibits high levels of intra-population genetic diversity. Whilst no obvious population genetic differentiation was identified by analysis of mtDNA sequences, pronounced population structure was observed based on microsatellite analysis. The gradient-like nature of the genetic differentiation (from west to east) observed for the nuclear DNA microsatellite strongly suggests that the result is real and not artefactual, despite the limited number of sites from which it was possible to collect fish. These analyses also clearly identified for the first time three genetically distinct subpopulations: west (SG), central (MG, QX and SN) and east (ML) within the YLTR. Additional sampling in the future from sites in the upstream (western) and downstream (eastern) regions of the YLTR will allow testing of both the major east-west differentiation and also of the three clades.

### Genetic diversity

Genetic diversity has been identified by the IUCN as an important form of biodiversity, deserving conservation within each population^27^. Our study, using both mitochondrial and nuclear markers, revealed that genetic variation is relatively high in *S. waltoni*, despite the threats this species presently faces. The mtDNA results (both genetic variation indices *Hd* and π) reveal that *S. waltoni* has high levels of genetic diversity comparable to other schizothoracine fishes found on or around the QTP^23,26,28^. However, when combined, the mtDNA results indicate that *S. waltoni* falls into a group with a high level of haplotype diversity (*Hd* > 0.5) and a moderate level of nucleotide diversity (*π* > 0.005)^29^, which is different from the genetic di**v**ersity pattern (high haplotype diversity but low nucleotide diversity) observed in other schizothoracine fishes^23,26,28^.

Due to the tetraploid inheritance pattern of schizothoracine fishes, few population genetic studies based on microsatellite markers for these fishes have been reported, meaning that judging absolute levels of nuclear DNA diversity is difficult. The mean number of bands per microsatellite locus (24.18) of *S. waltoni* falls between the two previous estimates with which comparison can be made^28,30^.

A high level of mtDNA haplotype diversity and a moderate level of nucleotide diversity in *S. waltoni* are indicative of either secondary contact between differentiated lineages or a large stable population with a long evolutionary history^29^. Differentiating between these two explanations is difficult. The QTP has undergone several periods of pronounced uplift since the Quaternary, creating substantial changes in the climate and the natural environment^31^ and significantly influencing the distributional ranges of schizothoracine fishes. Secondary contact of formerly isolated *S. waltoni* populations may therefore have occurred due to the uplift and climate change of the QTP. In support of the second explanation, we note that neutrality tests and mismatch distribution analyses confirmed the likely existence of a stable population with no recent expansion events for *S. waltoni*.

### Population genetic structure

Given their different evolutionary histories, mutation rates, modes of inheritance, and effective population sizes, mtDNA markers are expected to reflect more accurately the historical (ancestral) situation, whereas nuclear DNA markers are expected to better reflect the contemporary situation. Taken together, the results may be informative in terms of understanding how landscape changes (e.g., major and rapid uplift on the QTP resulting in isolation in a high altitude environment) have influenced the distributions over time of *S. waltoni* and its population genetic structure^23,32^.

Analysis of concatenated mtDNA sequence data indicated that there was moderate-high and significant genetic differentiation between ML (most easterly population) and the other four upstream populations (SG, MG, QX, SN) which formed an undifferentiated group. However, all five populations had private haplotypes which suggests that there must be some minor barrier to gene flow amongst populations to maintain this low level of differentiation over many generations. In the phylogenetic analyses, two clusters (evolutionary lineages) were identified. However, the haplotypes clustered in one subgroup were not from a specific population, but were from all five populations (Fig. 2; Table S1).

The shared mtDNA haplotypes and overlapping spatial distributions of the fishes in the two evolutionary lineages most probably reflect a scenario of secondary contact after a period of isolation. Historically, geological events and climate change have significantly influenced the diversity and population genetic structure of endemic organisms on the QTP^33,34^. Towards the eastern end of the YLTR, between Shannan and Mainling, there is the 30 m high Nierikar Waterfall (NW, meaning “fishes are blocked in here” in Tibetan). This waterfall, which is located at the Jiacha Gorge (3,300 m above sea level (asl)), impedes contemporary upstream migration of fishes. However, due to the several glacial lakes impounded by the moraine^34^, the Nierikar Waterfall might not have impeded past (historical) upstream dispersal of *S. waltoni*. In the late Pleistocene to early Holocene, these ancient glacial lakes were located at the main stream lying immediately upstream of the Tsangpo Great Gorge^35^. According to He and Chen^34^, the surface elevation of these ancient glacial lakes (3,800 to 3,530 m asl) exceeded the elevation of the Nierikar Waterfall (3,300 m asl). Therefore, the ancient lakes could have extended to the upper stream of the Jiacha Gorge and the Nierikar Waterfall observed today and would not have prevented the upstream dispersal of *S. waltoni* in the Pleistocene^34^. No major cascades or waterfalls are found along the YLTR between Shigatse (SG) and Shannan (SN), and thus there is no impediment to gene flow (connectivity) amongst the populations of *S. waltoni* within this ~300 km of the river. As a consequence of the river’s morphology and geological history, pronounced mtDNA genetic differentiation was observed between ML (downstream of the Nierikar Waterfall) and the four other populations. The divergence times amongst the main clades are correlated with later Kunlun-Huanghe Movement (about 0.6 Ma–1.1 Ma) and the Gonghe Movement (~0.15 Ma)^31^, results are consistent with the main divergence time reported for *S. o’connori* in the same region (0.30 Ma–0.43 Ma)^23^.

Pronounced population genetic structure of *S. waltoni* was identified by analysis of microsatellite variation which reflects contemporary (last few generations) gene flow. Consistent with the mtDNA results, the nuclear DNA results revealed significant genetic divergence between ML in the east and the four upstream (west) populations, and they also revealed small but significant genetic divergence between SG (most western population) and the three central populations (MG, QX, SN). Such discordance between nuclear and mtDNA markers is an oft reported phenomenon^36^. However, no obvious population genetic structure was found based on the analyses of 257 putatively neutral microsatellite bands. Thus, the genetic signal of differentiation is largely or solely contained within the nine bands that are under putative selection, highlighting the importance of retaining signal-bearing bands (alleles) under certain circumstances for quantification of spatial genetic differentiation^37,38^. We do, however, note that retention of bands that are putatively under selection will violate some assumptions of neutrality associated with many analyses. In addition, we note that the treatment of codominant microsatellite loci as dominant loci (allelic phenotypes were transformed into a presence/absence data set) is unusual but is warranted in the present case given the tetraploid nature of the focal species. Regardless, from a management perspective, the data set is informative because it provides evidence of spatial genetic structure that might otherwise have been overlooked.

The existence of two evolutionary lineages received strong support from the STRUCTURE and BAPS analyses of microsatellite variation, which showed that the SG (most westerly) and ML (most easterly) populations represent two different clusters of *S. waltoni*, whereas a mixed cluster was found in the three central populations (MG, QX, SN). These results were confirmed by the PCoA and by a significant pattern of IBD. This pronounced microsatellite population genetic structure reflects the contemporary erosion of genetic differences that accumulated when the original population of *S. waltoni* was split into two, and is, in effect, a secondary zone of contact between the two contemporary lineages. In terms of directional gene flow between the two lineages there are no substantial waterfalls along the YLTR in the region of the three central populations (MG, QX, SN) today, although several small waterfalls are found between SG and QX which may act to reduce or prevent gene flow and/or dispersal up the river (from QX and further upstream to SG in the west) but not in the other direction. These results indicate that waterfalls in the far eastern (near ML) and far western (near SG) regions, specifically between the populations SN and ML and between SG and QX, may block contemporary gene flow of *S. waltoni*, whereas contemporary gene flow is likely to be frequent amongst the three central populations where there are no waterfalls. Clearly, these findings and our interpretation of them, will benefit from further sampling of *S. waltoni* populations, both in the west, above the Nierikar Waterfall to test clade diversity, and in particular to the east, below the Nierikar Waterfall, from where we presently only have one population. Further sampling in the east will help to quantify just how widely distributed and abundant this fish is in a region that appears to be outside its main range of distribution.

### Conservation and management implications

Due to its *K*-selected life-history characteristics, *S. waltoni* is susceptible to growth overfishing and stock depletion^39^. Because of the inherent difficulties in studying *S. waltoni*, the biology of the species continues to be poorly understood, a fact that has hampered its effective conservation and allowed for conservation planning only on a local scale (i.e. the Lhasa Wild Fish Protection Regulation).

Fishes of the YLTR face a range of anthropogenic threats, including heavy fishing pressure, habitat loss/modification, climate change and introduced species. Of particular concern, however, is dam construction that is both an immediate and long-term threat to connectivity. The environmental impact of greatest concern is that the dam changes the flow of the river from an upstream river valley to a reservoir^40^. Additionally, there may be changes in the downstream water quality including river temperature, nutrient load, dissolved gases, heavy metals, and sediments^40^. Dams may cause habitat loss, change fish reproductive environments, and hinder breeding fish migration, resulting in a substantial decline of fish genetic resources^40–42^. In 2014, construction of the Zangmu Dam (with a fish ladder), the first dam on the main stream of the YLTR, was completed. Another seven (at least) dams will be built in the main stream of the YLTR in the future and all seven dams are to be located in the region between SN and ML. These dams will add to the existing natural barrier to fish movement that already exists in this region caused by the Nierikar Waterfall. Existing genetic differences between the lineages may be enhanced by the construction of dams that may further reduce or prevent downstream (i.e., SN to ML) movement of fishes even more than the existing Nierikar Waterfall. Although the dam plan includes fish ladders, the success of a fish ladder is unknown and will need to be evaluated over a long time to determine its success, given that even the most efficient protective measures cannot prevent some level of deterioration of ecosystem function^40,42^.

Evolutionarily significant units (ESUs) and management units (MUs) are two important concepts in conservation genetics^43^. An ESU is defined as a population that exchanges few migrants with others and that has been geographically isolated for long enough to be genetically distinct and independent^43^. High genetic differentiation (between ML and the four other populations) but no obvious population structure was observed in *S. waltoni* based on mtDNA analysis. However, moderate genetic differentiation and pronounced population structure are found based on microsatellite analysis. Because no reciprocal monophyly was found in this study, we propose a single ESU for the five populations of *S. waltoni*. Given the results of the microsatellite analyses, three groups ([SG], [MG + QX + SN], and [ML]) should be defined as different MUs worthy of protection as distinct stocks. However, *S. waltoni* collected from the western region (SG or MG) have been used as broodstock in the artificial reproduction programme at ML. The question arises whether these western parental fish are genetically similar to the local fish in ML and whether the juveniles (used for reintroduction downstream) hatched from the western parents are able to adapt to the area of their release (e.g. ML). Our results clearly show that western *S. waltoni* (fish from SG, MG, QX, SN) are genetically different from the fish at ML, in the east. As such, we suggest that it is appropriate to cancel, or at least postpone, the further collection of sexually mature *S. waltoni* from the western region to be used as broodstock for release of juveniles at ML until tools enabling the fitness of individuals to be determined in specific environments become available.

## Material and Methods

### Ethics statement

There were no specific permits required for the field research in this study. We confirm that there was no involvement of any endangered species in the field sampling. The fish sampling plan and animal research protocol were approved by the animal research oversight committee of Huazhong Agricultural University (HZAU). All experimental protocols were approved by the Institutional Animal Care and Use Committee of HZAU, and all methods were performed in accordance with relevant guidelines and regulations.

### Sample collection, sample sizes and DNA isolation

Sampling was designed to collect *S. waltoni* from as many sites as possible in the YLTR (Fig. 1) because *S. waltoni* is only found in the main channel and most tributaries (e.g. Lhasa River (LSR) and Nyang River (NYR)) of the YLTR above the Yarlung Tsangpo Grand Canyon^14^. However, in our sampling, it was difficult to find *S. waltoni* in the tributaries (e.g. Nyingchi along the NYR), and even in the main channel (e.g. Paizhen). Therefore, based on sampling success rather than on number of sites visited, fish were collected from five populations (Shigatse (SG), Maldrogongkar (MG), Quxu (QX), Shannan (SN) and Mainling (ML)) during 2012 to 2013 (Table S8). Based on their position relative to the 30 m high Nierikar Waterfall (Fig. 1), the populations were divided into western and eastern regions (west of NW: SG, MG, QX and SN; east of NW: ML). All fishes were captured by gillnetting or electro-fishing; fin clips were preserved in 95% ethanol at −20 °C.

Population-specific sample sizes reflect a balance across a range of different factors, including time/sampling effort, cost, information content of the marker itself, and the fact that *S. waltoni* is a threatened species that is difficult to capture and which now exists at low population numbers. Clearly sample size is important and will influence the results and their interpretation. For microsatellite analyses, 25 to 30 individuals per population is enough to accurately estimate allelic frequencies if locus-specific levels of polymorphism are not very high for this biparentally inherited nuclear DNA marker type^44^. For mtDNA, which is maternally inherited and does not experience recombination, it is generally accepted that half of the microsatellite sample size is enough for phylogeography and population genetics analyses. In this study, sample sizes for the microsatellite analyses averaged 44.2 and for the mtDNA averaged 22.8 individuals per population.

Genomic DNA isolation and concentration estimation were performed following Guo *et al*.^23^.

### mtDNA amplification, sequencing and data analysis

Complete cytochrome *b* (Cyt *b*, 1141 bp) and partial control region (CR, 712 bp) were sequenced according to Guo *et al*.^23^. Cyt *b* sequences were amplified using primers L14724 and H15915^34^, and CR sequences were amplified using DL-F and DL-R^23^. Cyt *b* sequences were sequenced in forward and reverse directions, CR sequences were sequenced in the forward direction.

Multiple sequence alignment, haplotype diversity (*Hd*), nucleotide diversity (*π*), number of segregating sites (*S*), number of haplotypes (*h*), number of nucleotide differences (*K*), median-joining network and Kimura 2-parameter (K2P) genetic distances between populations were performed/estimated following Guo *et al*.^23^. We focussed primarily on the concatenated sequence (Cyt *b* + CR) results because Cyt *b* + CR provided most information. Analysis of molecular variance (AMOVA) was carried out using Arlequin 3.5^45^ to test for genetic differentiation amongst populations/regions. 10,000 permutations were performed to examine statistical significance of fixation indices (*Φ*). Correction for multiple testing was performed using the Benjamini-Yekutieli method false discovery rate (BY-FDR)^46^, and comparisons were considered significant at *P* < 0.05.

To examine relationships amongst populations and to test for evidence of historical dispersal, phylogenetic analysis was employed based on Cyt *b*, CR and the concatenated data set (Cyt *b* + CR) independently using neighbour-joining (NJ) and maximum likelihood (ML) in MEGA 5.0^47^. Except for the HKY + G (Cyt *b*), T92 + G (CR) and HKY + G (Cyt *b* + CR) models used for ML analyses, the other procedures of phylogenetic analysis were carried out following Guo *et al*.^23^. *S. o’connori* (GenBank accession no. KC513575), *S. macropogon* (KC020113) and *Schizopygopsis younghusbandi* (KC351895) were used as outgroups.

Bayesian phylogenetic analyses were performed to build Markov chain Monte Carlo (MCMC) trees with Cyt *b* and the concatenated mtDNA data (Cyt *b* + CR) independently following Guo *et al*.^23^, and divergence time amongst the main clades were added on the MCMC trees. Coalescent-based Bayesian skyline plots (BSPs) and mismatch distributions were used to estimate historical population dynamics. BSPs were carried out following Guo *et al*.^23^ to estimate the time variation of effective population size to determine demographic history. Mismatch distributions^48^ were implemented in Arlequin 3.5 and DnaSP 5.1^49^. Tajima’s *D* and Fu’s *F*s tests were performed to test if the two mtDNA sequences accorded with the expectations of neutrality^50,51^. The sum of squared deviations (*SSD*) and the raggedness index (*r*) were calculated using Arlequin 3.5 to test if sequences deviated significantly from a population expansion model^52^. All *P*-values were corrected using the BY-FDR. The population expansion time in years (T) was estimated as described by Guo *et al*.^23^. The generation time for *S. waltoni* was estimated to be 10 years based on age of female maturation^18^. We only considered female age due to the maternal inheritance of mitochondrial DNA. Mutation rates of Cyt *b*, CR and Cyt *b* + CR were assumed to be 1.0%, 3.6% and 1.69% per site per million years, respectively^23^.

### Nuclear microsatellite genotyping and data analysis

Eleven microsatellite markers^23^ were selected based on allelic size range, annealing temperature and scoring performance. Primers (Table S3) were synthesised and labelled with fluorescent dyes at the 5′ ends for PCR amplification^23^. Capillary separation and allele size scoring were performed following Wei *et al*.^37^ and Guo *et al*.^23^. Because *S. waltoni* is a tetraploid fish, and because it is unknown which alleles form pairs, we were unable to use software such as LOSITAN^53^ to test for outlier loci. Therefore, microsatellite loci were considered as dominant markers and multilocus allelic phenotypes were transformed into presence (1) or absence (0) for each individual. Each band was considered as a single bi-allelic locus with an amplifiable (dominant) and a null (recessive) allele. To detect candidate bands under selection, the *F*_ST_ outlier method was performed using three softwares: BayeScan 2.1^54^, Arlequin 3.5 and Mcheza^55^. For BayeScan, we used the default values with respect to the thinning interval, tuning of the proposal distributions, the number of pilot runs, and the uniform prior boundaries for *F*_IS_ and included a burn-in period of 50,000 iterations and prior odds of the neutral model of 10. Default settings were also used in the Arlequin and Mcheza analyses. The data set was divided in two subgroups, “neutral” and “selected” (outlier) loci following Luikart *et al*.^56^. For the neutral data set, we removed the bands putatively identified as outliers, both those classified as under directional and stabilizing selection, by any two of the three methods (BayeScan, Arlequin and Mcheza). All analyses were performed on the all bands and the neutral bands data sets.

Genetic diversity indices such as Nei’s gene diversity index (*H*), Shannon’s information index (*I*), the percentage of polymorphic loci (*PPL*), Nei’s unbiased genetic distance, and the relationships between populations and individuals (PCoA) were evaluated following Guo *et al*.^23^. Using the binary data set, a Bayesian clustering analysis was carried out in STRUCTURE 2.3^57^ to determine the expected number of genetic clusters, as described by Guo *et al*.^23^. Ten independent runs were performed for each cluster set (*K*) from 1 to 5. The Δ*K* metric^58^ was used to assess the best fitting *K* through Structure Harvester (http://taylor0.biology.ucla.edu/structureHarvester/) and runs were grouped and visually displayed using CLUMPAK (http://clumpak.tau.ac.il/). To confirm the distribution of individuals amongst genetic groups and verify the adscription of individuals to the clusters revealed by STRUCTURE, we also used the clustering approach implemented in BAPS 6.0^59,60^. BAPS analyses were carried out on groups of individuals (i.e. site-samples) rather than individuals, with simple model assumptions (i.e. no admixture and uncorrelated allele frequencies). BAPS was set with 10 replicate runs for each *K* (from 2 to 5) and another 100 replicate runs with *K* fixed to the inferred number of genetic clusters (best *K*). The best fitting *K* was assessed using the highest Log marginal likelihood [Log(ML)] value. And then, an admixture model was run for the inferred number of clusters (1000 iterations, minimum individuals per cluster = 5, reference individuals = 200, iterations for reference individuals = 10).

### Isolation by distance (IBD)

We tested for IBD using GENEPOP (http://genepop.curtin.edu.au/) to investigate whether genetic distance ([*Φ*_ST_/(1 − *Φ*_ST_)] for mtDNA data or [*F*_ST_/(1 − *F*_ST_)] for microsatellite data) was correlated with geographic distance [Ln(*d*)] amongst the five populations (Table S9), as recommended for a two-dimensional model^61^. Significance levels were determined using separate Mantel tests for the mtDNA and microsatellite data sets as implemented in ISOLDE by GENEPOP employing 20,000 permutations.

## Supplementary information

Supplementary information for phylogeography of <i>Schizothorax waltoni</i>
