# Supplementary material for: Phylogeography of the threatened tetraploid fish, Schizothorax waltoni, in the Yarlung Tsangpo River on the southern Qinghai-Tibet Plateau: implications for conservation
Source: Sci Rep. 2019 Feb 25;9:2704. doi: 10.1038/s41598-019-39128-y (PMC6390103; doi:10.1038/s41598-019-39128-y)
Supplement: Supplementary file 1 — Supplementary information for phylogeography of Schizothorax waltoni [file 41598_2019_39128_MOESM1_ESM.pdf]

Phylogeography of the threatened tetraploid fish, *Schizothorax waltoni*, in the Yarlung Tsangpo River on the southern Qinghai-Tibet Plateau: implications for conservation

Xiang-Zhao Guo[1, 2, †], Gui-Rong Zhang[1, 2], Kai-Jian Wei[1, 2, *], Wei Ji[1, 2], Ruo-Jin Yan[1, 2, 3], Qi-Wei Wei[4] and Jonathan P. A. Gardner[1, 2, 3]

[1] - Key Laboratory of Freshwater Animal Breeding, Ministry of Agriculture, College of Fisheries, Huazhong Agricultural University, Wuhan 430070, P. R. China

[2] - Freshwater Aquaculture Collaborative Innovation Center of Hubei Province, Wuhan 430070, P. R. China

[3] - School of Biological Sciences, Victoria University of Wellington, P O Box 600, Wellington 6140, New Zealand

[4] - Key Laboratory of Freshwater Biodiversity Conservation, Ministry of Agriculture, Yangtze River Fisheries Research Institute, Chinese Academy of Fishery Sciences, Wuhan 430223, P. R. China

[†] - Present address: Guangdong Haid Group Co., Ltd., Guangzhou 511400, P. R. China

* - **Correspondence**:

Kai-Jian Wei, College of Fisheries, Huazhong Agricultural University, Wuhan 430070, P. R. China. Tel: 86 27 87282113, Fax: 86 27 87282114. E-mail: kjwei@mail.hzau.edu.cn

Supplementary information

Figure S1. The neighbour-joining phylogenetic tree of *Schizothorax waltoni*, based on mtDNA Cyt *b* haplotypes. The haplotypes provided at each twig and their geographical locations are shown in Table S1 and Fig. 1. The numbers above the branches correspond to bootstrap support values > 50% obtained in the NJ / ML analyses, respectively.

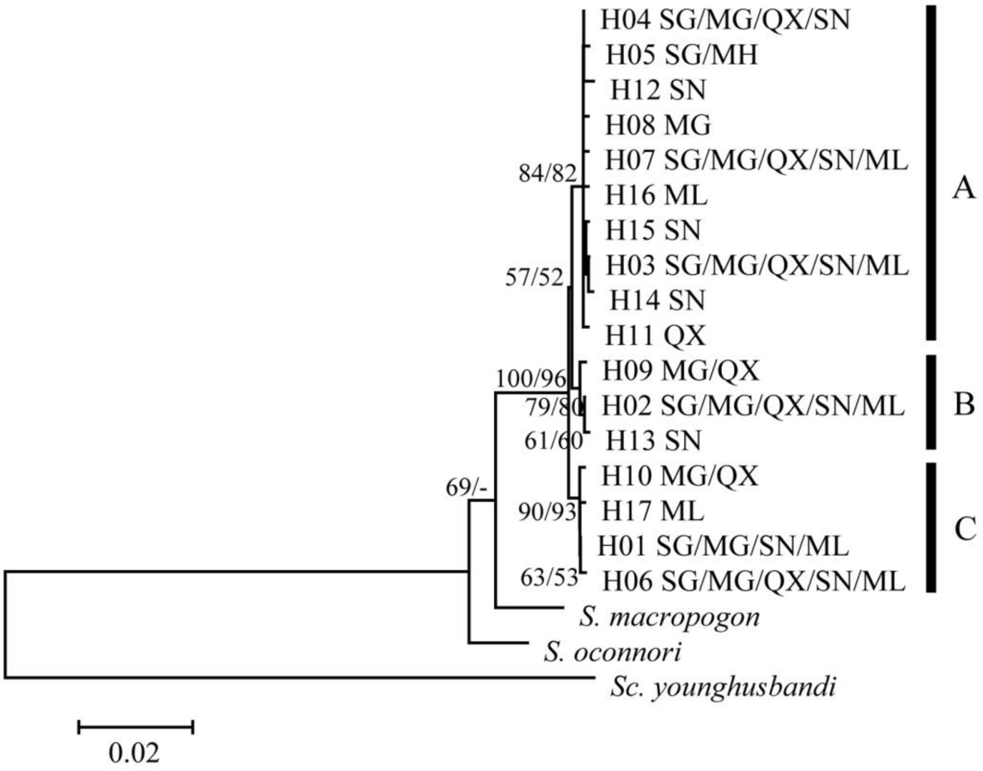

Figure S2. The neighbour-joining phylogenetic tree of *Schizothorax waltoni*, based on mtDNA CR haplotypes. The haplotypes provided at each twig and their geographical locations are shown in Table S1 and Fig. 1. The numbers above the branches correspond to bootstrap support values > 50% obtained in the NJ / ML analyses, respectively.

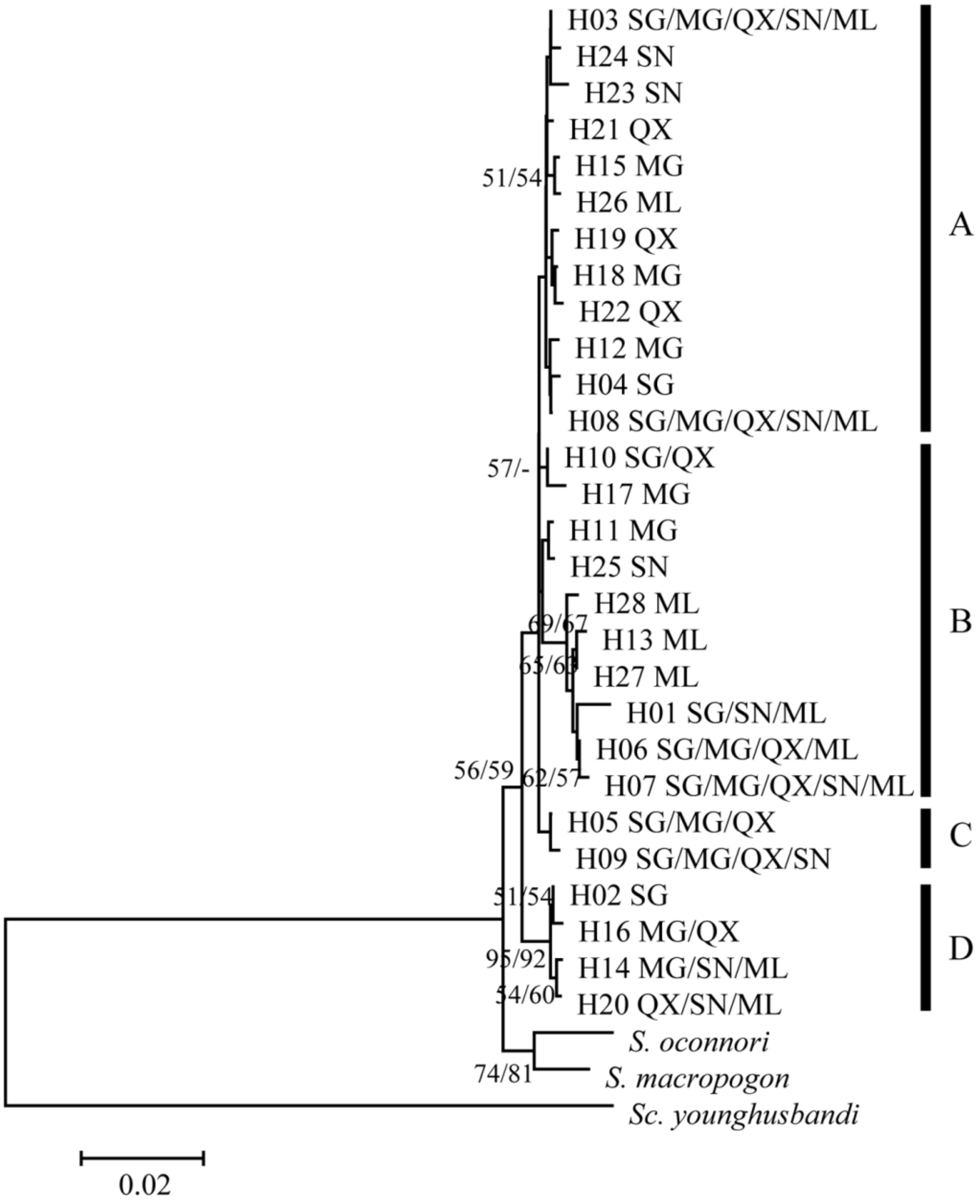

Figure S3. Bayesian phylogenetic tree for *Schizothorax waltoni*, based on mtDNA Cyt *b* haplotypes. The numbers above the branches are the estimates of divergence times (million years, Ma) within *Schizothorax waltoni* for the major nodes by BEAST analysis. Blue shaded bars indicate the 95% highest posterior density (HPD) for node ages and scale bar represents time in millions of years from the present day.

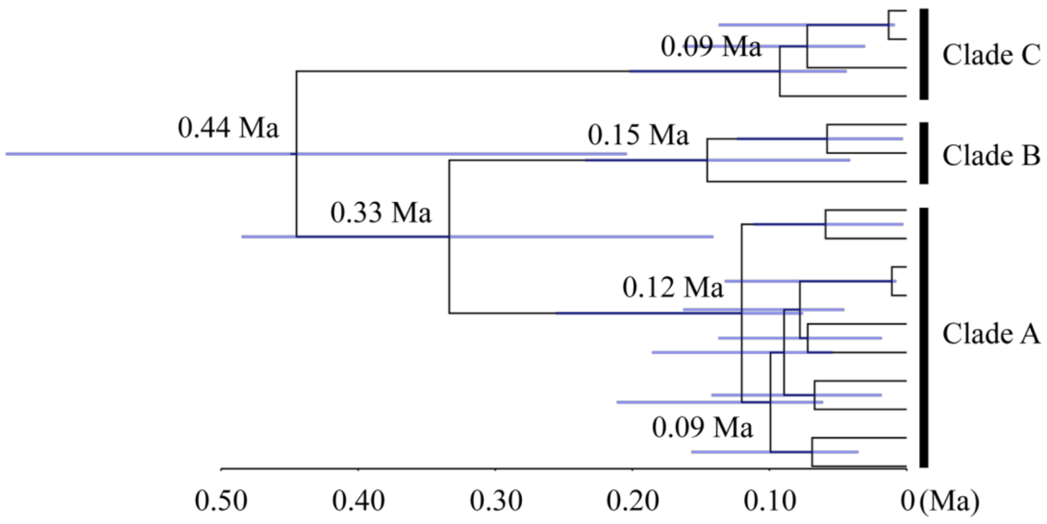

Figure S4. Bayesian skyline plots (BSPs) of *Schizothorax waltoni*. (a) clade A of Cyt *b*; (b) clades A+B of Cyt *b*; (c) clade A of Cyt *b*+CR; (d) clades A+B of Cyt *b*+CR. The *X*-axis shows time in millions of years before present. The *Y*-axis (logarithmic scale) indicates effective population size of females (Ne) estimates multiplied by generation time (T_{gen}). The solid line indicates the median of population size, and the 95% highest posterior density (HPD) interval is depicted in blue.

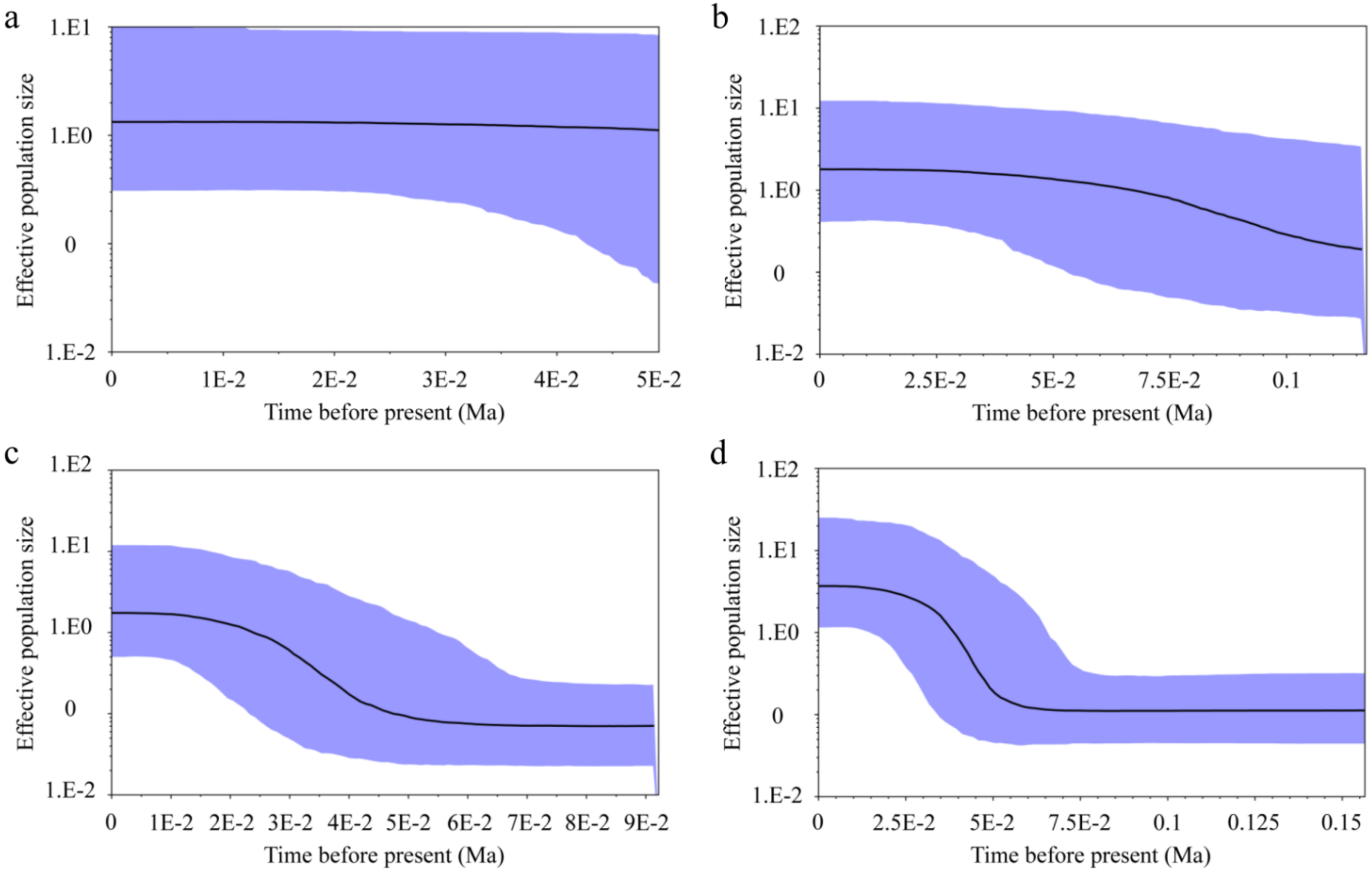

Figure S5. Plots of potential phenotypic loci of microsatellites under selection detected by BayeScan 2.1 (a), Arlequin 3.5 (b) and Mcheza (c)**.** In (a): Plots of F_{ST} values against logrithm of posterior odds $\log_{10}$(PO); each black dot represents an microsatellite band, vertical line represents the threshold of used for identifying outlier loci, the 11 black dots on the right side of the vertical line represent potential loci under selection. In (b): Distribution of F_{ST} values as a function of locus heterozygosity; each dot represents a microsatellite band; the blue dots, red dots and black circles represent locus beyond 99% confidence interval (CI), within 95% – 99% CI and within 95% CI, respectively; the red dots above the upper red dashed line are classified as outliers potentially under positive selection; the red dashed line, blue dashed line, black dashed line and black solid line represent 99%, 95%, 90% and 50% CI, respectively; (c) Distribution of F_{ST} values as a function of locus heterozygosity; each dot represents a microsatellite band; loci in the gray area, yellow area and red area are candidates under balance selection, neutral loci and positive selection; the gray area, yellow area and red area represent the area beyond upper 99% CI, within 99% CI and beyond lower 99% CI, respectively.

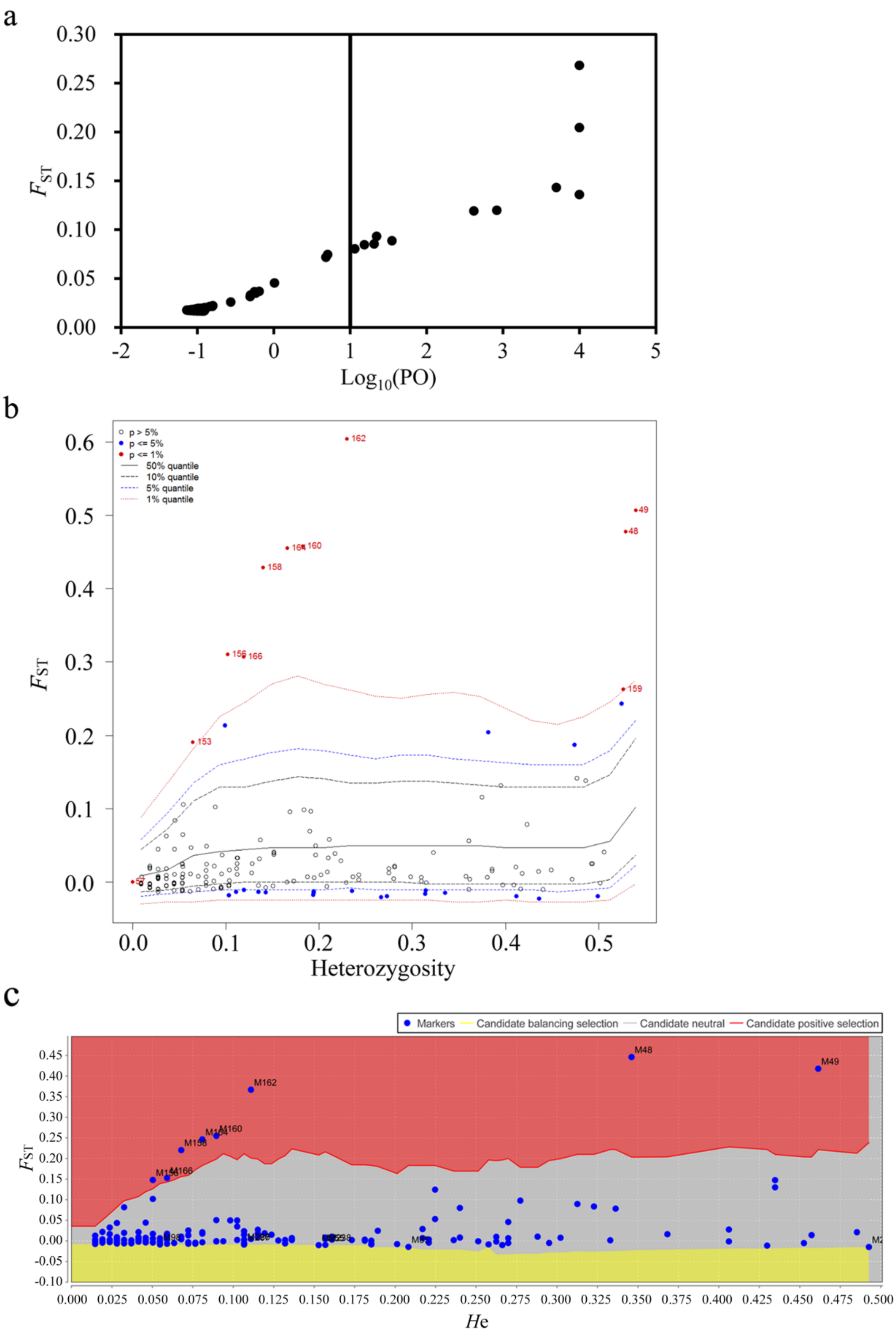
a
F_{ST}
Log10(PO)
b
p > 5%
p <= 5%
p <= 1%
50% quantile
10% quantile
5% quantile
1% quantile
162
49
48
160
158
156
166
159
153
F_{ST}
Heterozygosity
c
Markers
Candidate balancing selection
Candidate neutral
Candidate positive selection
M48
M49
M162
M160
M158
M166
F_{ST}
He

Figure S6. Cluster analyses of *Schizothirax waltoni* populations based on neutral amplified bands at 11 microsatellite loci. (a) Inference of best *K* in STRUCTURE 2.3; (b) Inference of best *K* in BAPS 6.0; (c) Histogram of the assignment test using STRUCTURE 2.3 (*K* = 2); (d) Histogram of the assignment test using BAPS 6.0 (*K* = 2); Each individual is represented by a vertical coloured line, and each color corresponds to a genetic cluster.

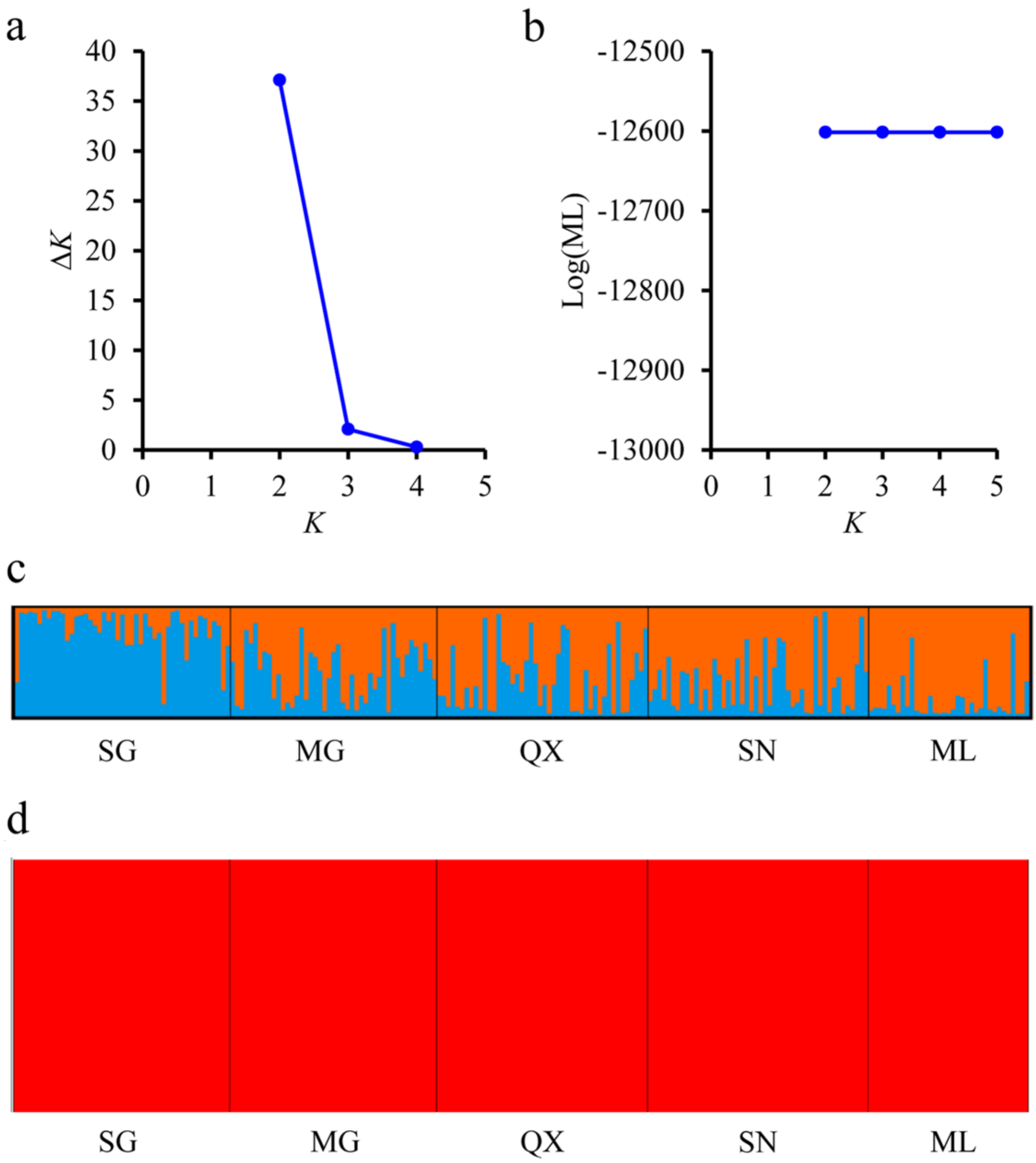

Figure S7. Genetic relationship amongst five populations of *Schizothorax waltoni* based on neutral amplified bands at 11 microsatellite loci. (a) NJ clustering tree based on the Nei's unbiased genetic distances amongst populations; (b) Principal coordinate analysis (PCoA) based on the pairwise genetic distances between individuals; (c) PCoA based on the pairwise genetic distances between populations.

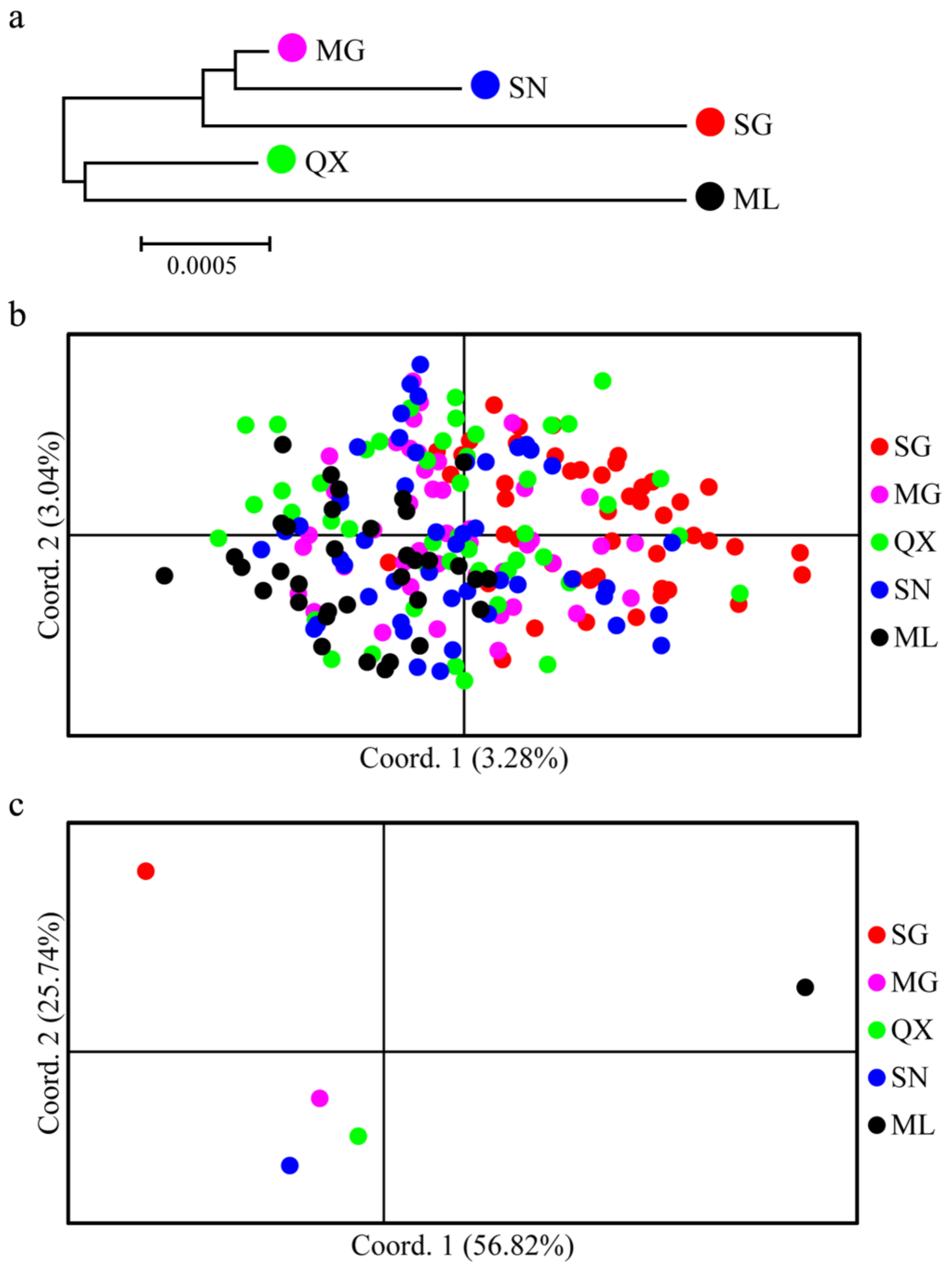

Figure S8. Scatterplots of genetic distance and geographic distance for pairwise population comparisons. The graph shows the linear regression of the mtDNA genetic distance (a) measured as $\Phi_{ST}/(1-\Phi_{ST})$ and microsatellite genetic distance (b) measured as $F_{ST}/(1-F_{ST})$ (based on 266 microsatellite bands) over logarithms of the geographic distance (in km). Dashed and solid lines represent non-significant (a, R^2 = 0.59, P = 0.097) and significant regressions (b, R^2 = 0.74, P = 0.043), respectively.

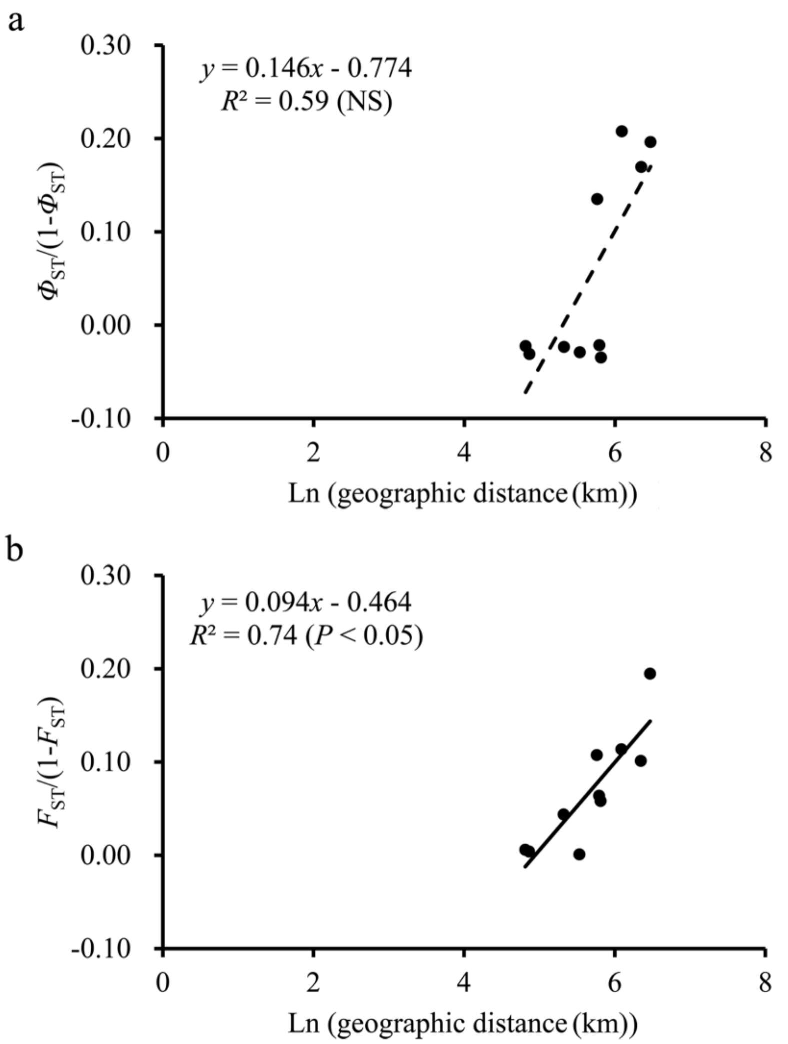

Figure S9. Scatterplot of genetic distance and geographic distance for pairwise population comparisons based on 257 neutrality microsatellite bands. The graph shows the linear regression of the microsatellite genetic distance measured as $F_{ST}/(1-F_{ST})$ over logarithms of the geographic distance (in km). Dashed lines represent a non-significant regression (R^2 = 0.69, P = 0.078).

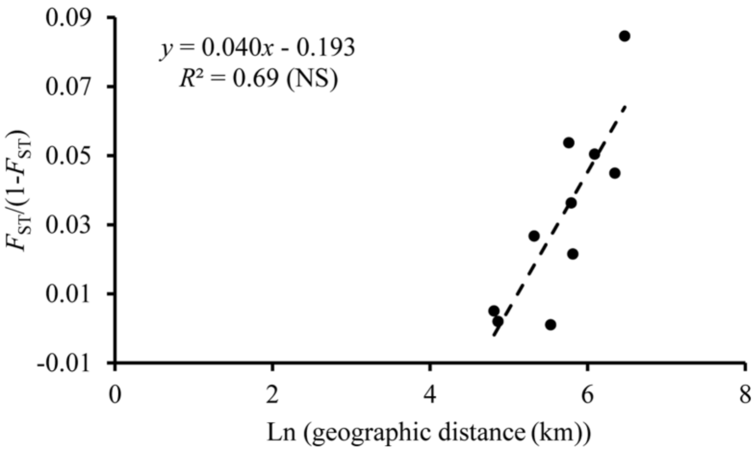

Table S1. Haplotype distributions in five populations of *Schizothorax waltoni* based on variation in the Cyt *b*, CR and concatenated Cyt *b*+CR sequence data set.

Sequence	Population	Haplotype (individual numbers)
Cyt *b*	SG	H01(3), **H02(3)**, **H03(4)**, H04(4), H05(3), **H06(1)**, **H07(5)**
	MG	H01(1), **H02(1)**, **H03(4)**, H04(5), H05(2), **H06(2)**, **H07(2)**, ***H08(1)***, H09(1), H10(1)
	QX	**H02(2)**, **H03(6)**, H04(8), **H06(2)**, **H07(1)**, H09(2), H10(1), ***H11(1)***
	SN	H01(1), **H02(2)**, **H03(6)**, H04(4), **H06(4)**, **H07(2)**, ***H12(2)***, ***H13(1)***, ***H14(1)***, ***H15(1)***
	ML	H01(8), **H02(4)**, **H03(4)**, **H06(5)**, **H07(1)**, ***H16(1)***, ***H17(1)***
CR	SG	H01(2), ***H02(3)***, **H03(4)**, ***H04(1)***, H05(4), H06(1), **H07(1)**, **H08(5)**, H09(1), H10(1)
	MG	**H03(3)**, H05(4), H06(1), **H07(2)**, **H08(1)**, H09(1), ***H11(1)***, ***H12(1)***, ***H13(1)***, H14(1), ***H15(1)***, H16(1), ***H17(1)***, ***H18(1)***
	QX	**H03(4)**, H05(2), H06(1), **H07(2)**, **H08(3)**, H09(3), H10(1), H16(2), ***H19(1)***, H20(2), ***H21(1)***, ***H22(1)***
	SN	H01(1), **H03(5)**, **H07(4)**, **H08(6)**, H09(2), H14(2), H20(1), ***H23(1)***, ***H24(1)***, ***H25(1)***
	ML	H01(1), **H03(4)**, H06(5), **H07(5)**, **H08(1)**, H14(2), H20(2), ***H26(1)***, ***H27(2)***, ***H28(1)***
Cyt *b*+CR	SG	H01(2), ***H02(3)***, **H03(1)**, H04(3), **H05(5)**, **H06(4)**, H07(1), H08(1), H09(1), ***H10(1)***, H11(1)
	MG	**H03(2)**, H04(2), **H05(1)**, **H06(3)**, H07(1), H09(2), ***H12(1)***, H13(1), ***H14(1)***, ***H15(1)***, H16(1), ***H17(1)***, ***H18(1)***,

	H19(1), H20(1)
QX	**H03(2)**, **H05(1)**, **H06(4)**, H07(3), H08(1), H09(2), H13(2), H16(1), ***H21(1)***, H22(2), ***H23(1)***, H24(1), ***H25(1)***, ***H26(1)***
SN	H01(1), **H03(3)**, **H05(2)**, **H06(4)**, H07(2), H20(1), H22(1), H24(2), ***H27(2)***, ***H28(1)***, ***H29(1)***, ***H30(1)***, ***H31(1)***, ***H32(1)***, ***H33(1)***
ML	H01(1), **H03(5)**, **H05(1)**, **H06(4)**, H11(4), H20(2), H22(2), ***H34(1)***, ***H35(2)***, ***H36(1)***, ***H37(1)***

Bold indicates haplotypes that are shared by all the five populations; bold italic indicates private haplotypes.

Table S2. Mismatch distribution and neutrality test for five populations of *Schizothorax waltoni* based on the Cyt *b*, CR and concatenated Cyt *b*+CR sequence data set.

Sequence	Population / haplotype clade in NJ tree	*SSD*	*r*	Distribution	*D*	*Fs*
Cyt *b*	SG	**0.094**	**0.188**	multimodal	0.242	0.790
	MG	**0.072**	0.121	multimodal	-0.567	-2.053
	QX	0.063	0.094	multimodal	-0.404	-0.146
	SN	0.043	0.066	multimodal	0.302	-0.961
	ML	**0.132**	**0.232**	multimodal	0.575	1.422
	Total	0.081	0.140	multimodal	0.030	-0.190
	Clade A	0.084	0.257	unimodal	-1.473	**-10.251**
	Clade B	0.042	0.222	unimodal	0	-0.693
	Clade C	0.122	0.500	unimodal	-0.754	**-2.367**
CR	SG	0.042	0.051	multimodal	0.030	-0.253
	MG	0.021	0.024	multimodal	-0.567	-4.748
	QX	0.025	0.030	multimodal	0.536	-1.753
	SN	0.023	0.029	multimodal	-0.085	0.084
	ML	0.018	0.026	multimodal	0.162	0.148
	Total	0.026	0.032	multimodal	0.015	-1.305
	Clade A	0.032	0.127	unimodal	-1.121	**-12.849**
	Clade B	0.016	0.040	unimodal	-0.500	**-5.879**
	Clade C	0	0	unimodal	0	0
	Clade D	0.073	0.333	unimodal	0.168	**-2.181**
Cyt *b*+CR	SG	**0.942**	0.085	multimodal	0.122	0.804
	MG	0.059	0.043	multimodal	-0.596	-3.318

QX	0.057	0.035	multimodal	0.113	-1.438
SN	**0.359**	0.024	multimodal	0.078	-1.574
ML	0.024	0.022	multimodal	0.350	1.407
Total	0.288	0.042	multimodal	0.013	-0.824
Clade A	0.009	0.040	unimodal	**-1.726**	**-25.627**
Clade B	0.047	0.062	multimodal	-1.377	**-5.771**
Clade C	0.047	0.180	multimodal	-0.668	**-2.517**

SSD, sum of the squared differences under expansion model; *r*, raggedness index; Distribution, the shape of mismatch distribution; *D*, Tajima's *D* test statistic; *Fs*, Fu's *Fs* test statistic. Significant values are in bold after FDR testing ($P < 0.05$).

Table S3. Details of the 11 microsatellite primers used for genotyping.

Locus name	GenBank accession no.	Primer sequence (5'-3') (F, forward; R, reverse)	Repeat motif	T_a (°C)	Multiplex	Fluorescent labelling	N_A	N_P	Band size range (bp)
JLL 01	KC880056	F: TCATTTACACAGTAGGGAGC	$(AC)_4…(TCCTC)_4$	54	3	TAMRA	23	1 – 4	207 – 267
		R: CAGTTAGAGGTGACGGAAG							
JLL 21	KC880076	F: GACAGACAGAAAGACCAGAGA	$(AGAT)_{12}$	56	3	FAM	16	1 – 4	102 – 159
		R: GGTAAACTATCCCAAAATCAT							
LLK27	KC907359	F: ATCATTCAAAGGTCACTCGT	$(TAGA)_8$	58	4	FAM	15	1 – 4	137 – 162
		R: TCCACAGAGATGCCAAAG							
LLK28	KC907360	F: GAACGAGAAAGTTAAAGGTC	$(ATAG)_{21}$	55	3	HEX	5	1 – 3	175 – 195
		R: AGGAGTGGTCAGTGCTTC							
Scho01	KC247930	F: TAATGATAATGCCGTGTCGTA	$(TG)_{12}$	57	2	HEX	22	1 – 4	247 – 293
		R: GAAACAGAAAACAGCCCAGAT							
Scho23	KC902766	F: CACACAATCAGTAGGTCAGG	$(AGAC)_6…(TG)_6$	60	1	FAM	5	1 – 3	238 – 250
		R: ACTAGCAGTTTATCTTCTCAGC							
Scho24	KC902767	F: ATTTTTCCTCTGCCCATTGA	$(CTAT)_{17}…(GTCT)_8…(GTCT)_9$	56	2	FAM	62	1 – 4	165 – 357
		R: TTGTGAACCGTTACACCCCT							

Scho26	KC902769	F: GCAAAGCACAAAGGATCT	$(TCTG)_4$…$(TCTA)_7$	58	1	HEX	25	1 – 4	103 – 158
		R: CTGAACCATTACACCCCTA							
Scho27	KC902770	F: CGTCTATTGTCTGCTCATCA	$(ATAG)_{14}$	56	1	TAMRA	34	1 – 4	119 – 198
		R: ATCTGCTTACGCCCCAT							
Scho40	KC902783	F: TAGAGGAGGATGGGTGAGAA	$(TCTA)_9$	54	4	TAMRA	26	1 – 4	213 – 300
		R: CCAACACTGCGAACGATAG							
Scho42	KC902785	F: ATAAGAGGAAAACAATGCC	$(GATA)_{17}$	56	4	HEX	31	2 – 4	143 – 233
		R: AGACCAATGTGTAACAGTAATG							

T_a, annealing temperature of each primer; N_A, number of bands observed at each locus; N_P, number of bands observed in each tetraploid individual.

Table S4. Lists of potential phenotypic loci of microsatellites under selection detected by three methods.

BayeScan 2.1		Arlequin 3.5		Mcheza	
Phenotypic locus*	Microsatellite marker	Phenotypic locus*	Microsatellite marker	Phenotypic locus*	Microsatellite marker
–	–	–	–	M26	JLL21
–	–	*M48*	LLK27	*M48*	LLK27
–	–	*M49*	LLK27	*M49*	LLK27
M50	LLK27	–	–	–	–
M51	LLK27	–	–	–	–
M53	LLK27	–	–	–	–
–	–	M57	LLK28	–	–
–	–	–	–	M85	Scho23
–	–	–	–	M98	Scho24
–	–	M153	Scho26	–	–
–	–	*M156*	Scho26	*M156*	Scho26
M157	Scho26	–	–	–	–
**M158**	Scho26	**M158**	Scho26	**M158**	Scho26
M159	Scho26	*M159*	Scho26	–	–
**M160**	Scho26	**M160**	Scho26	**M160**	Scho26
M161	Scho26	–	–	–	–
**M162**	Scho26	**M162**	Scho26	**M162**	Scho26
M163	Scho26	–	–	–	–
**M164**	Scho26	**M164**	Scho26	**M164**	Scho26
–	–	*M166*	Scho26	*M166*	Scho26
–	–	–	–	M226	Scho40
–	–	–	–	M238	Scho42
–	–	–	–	M255	Scho42
–	–	–	–	M257	Scho42

* Potential phenotypic loci of alleles under selection; Bold indicates the phenotypic loci were identified by all three methods; Italic indicates the phenotypic loci were identified by two methods.

Table S5. Nuclear DNA diversity indicies for five populations of *Schizothorax waltoni* based on 257 neutrality microsatellite bands.

Population	Total bands	Private bands	*PPL* (%)	*H*	*I*
SG	171	24	65.76	0.080	0.143
MG	177	15	67.70	0.080	0.146
QX	156	5	59.92	0.077	0.138
SN	164	10	63.04	0.077	0.139
ML	128	11	49.03	0.067	0.119
Mean	159	13	61.09	0.076	0.137
Total	257	65	–	–	–

PPL, percentage of polymorphic loci; *H*, Nei's gene diversity index; *I*, Shannon's information index.

Table S6. Matrix of pairwise F_{ST} values (below diagonal) and Nei's unbiased genetic distances (above diagonal) amongst five *Schizothorax waltoni* populations based on variation at 257 neutrality microsatellite bands.

Population	SG	MG	QX	SN	ML
SG	–	0.002	0.003	0.003	0.005
MG	**0.021**	–	0.002	0.001	0.003
QX	**0.026**	0.002	–	0.002	0.003
SN	**0.035**	0.001	0.005	–	0.004
ML	**0.078**	**0.043**	**0.048**	**0.051**	–

Significant F_{ST} values are in bold after FDR testing ($P < 0.05$).

Table S7. Analysis of molecular variance (AMOVA) of *Schizothorax waltoni* population genetic variation based on 257 neutrality nuclear DNA microsatellite bands variation.

Source of variation	df	Variance component	Percentage of variation	Fixation index
Among populations	4	0.444	2.84	F_{ST} = 0.028***
Within populations	216	15.148	97.16	–
Between regions (west/east)	1	0.577	3.61	F_{CT} = 0.036
Among populations within regions	3	0.251	1.57	F_{SC} = 0.016***
Within populations	216	15.148	94.82	F_{ST} = 0.052***

***P< 0.001.

Table S8. Geographic locations, geospatial information and sample sizes for five *Schizothorax waltoni* populations.

Population	Code	Alt (m)	Lat	Long	n	N
Shigatse	SG	3842	29.32	88.86	23	47
Maldrogongkar	MG	3823	29.84	91.68	20	45
Quxu	QX	3595	29.35	90.73	23	46
Shannan	SN	3554	29.26	91.82	24	48
Mainling	ML	2964	29.19	94	24	35
Mean	—	—	—	—	22.8	44.2
Total	—	—	—	—	114	221

Alt, altitude (m); Lat, latitude (°N); Long, longitude (°E); n, number of individuals for mtDNA analysis; N, number of individuals for microsatellite analyses.

Table S9. Matrix of pairwise population actual geographical distances along the river course (km).

Population	SG	MG	QX	SN	ML
SG	–				
MG	334.39	–			
QX	204.87	129.52	–		
SN	327.93	252.58	123.06	–	
ML	645.98	570.63	441.11	318.05	–
